# P2RX7 regulates tauopathy progression via tau and mitochondria loading in extracellular vesicles

**DOI:** 10.21203/rs.3.rs-6771517/v1

**Published:** 2025-06-23

**Authors:** Tsuneya Ikezu, Victor Bodart-Santos, Arun Reddy Ravula, Yang You, Mohammad Abdullah, Zhi Ruan, Justice Ellison, Stephanie Radhakishun, Nibedita Basu Ray, Bridgette Melvin, Aishe Kurti, Zhewei Liang, Yuta Sakai, Daniel O’Brien, Son Nguyen, Scott Shaffer, Lindsey Fiddes, Oleg Butovsky, Wolfdieter Springer, Seiko Ikezu

**Affiliations:** Mayo Clinic Florida; Mayo Clinic; Mayo Clinic Florida; Mayo Clinic Floria; Mayo Clinic Florida; Mayo Clinic Florida; Mayo Clinic Florida; Mayo Clinic Florida; Mayo Clinic Florida; Mayo Clinic Florida; Mayo Clinic Jacksonville; Mayo Clinic; Mayo Clinic Rochester; Mayo Clinic; University of Massachusetts Chan Medical School; UMASS Chan Medical School; University of Toronto; Ann Romney Center for Neurologic Diseases, Department of Neurology, Brigham and Women’s Hospital, Harvard Medical School; Mayo Clinic Florida; Mayo Clinic Florida

**Keywords:** Alzheimer’s disease, extracellular vesicles, microglia, microtubule-associated protein tau, mitochondria, tauopathy, P2X purinoceptor 7

## Abstract

P2x purinoreceptor 7 (P2RX7), an ATP-gated ion channel, is known to play pivotal roles in the progression of Alzheimer’s disease (AD), although its cell type-specific pathological mechanisms have yet to be elucidated. Here, we show that genetic deletion of P2rx7 mitigates brain atrophy, tau accumulation and cognitive impairment in PS19 tauopathy mice. Specific deletion of P2rx7 in microglia, but not astrocytes, significantly suppresses tau propagation from the entorhinal cortex to CA1 in the hippocampus, an early event in AD pathology. Single-cell (sc)-RNA sequencing of mouse brains revealed specific P2rx7 expression in microglia, inducing inflammatory changes accompanied by elevated extracellular vesicles (EVs) secretion in PS19 mice. Brain-derived EVs (BDEVs) proteome demonstrated that P2RX7 increases EV cargo loading of tau and mitochondrial molecules in BDEVs from PS19 mice, which was further validated by single-molecule super-resolution. Notably, following the injection of BDEVs isolated from PS19 mice with or without P2rx7 deficiency, the microglial transcriptome of recipient mice revealed enriched DNA-sensing and type II interferon signaling in response to BDEVs from PS19 mice, which was diminished in the group injected with P2rx7-deficient BDEVs. Thus, our results indicate that P2RX7 regulates EV-mediated tau and mitochondrial transfer and inflammatory activation in microglia with increased EV secretion, thereby contributing to tauopathy and neurodegeneration, highlighting the therapeutic potential of targeting the P2RX7-EV axis in AD.

Alzheimer’s disease (AD) is a neurodegenerative disease characterized by extracellular amyloid beta (Aβ) plaques and intraneuronal hyperphosphorylated tau aggregates, known as neurofibrillary tangles (NFTs)^1^. While Aβ deposition occurs early and persists throughout the disease, tau pathology more closely correlates with cognitive decline and neurodegeneration^2, 3^. Growing evidence also links neuroinflammation—driven by Aβ and tau accumulation—to AD progression, and the identification of AD risk genes linked to innate immunity implicates glial cells as key contributors to disease progression^4, 5^.

Microglia, the innate phagocytic cells of the central nervous system (CNS), are crucial for maintaining homeostasis and mediating pathological signals in the brain^6, 7^. Upon activation by phagocytosis or extracellular stimuli such as extracellular ATP (eATP) released by damaged cells, microglia secrete cytokines and other mediators, including extracellular vesicles (EVs), contributing to neuroinflammation^4, 8, 9^. EVs are membranous structures involved in cellular communication, facilitating the transfer of biologically active molecules such as proteins, RNA, lipids, and metabolites between cells^10, 11^.

In AD and other neurodegenerative diseases, EVs spread pathological proteins^12, 13^. We recently identified tau in EVs isolated from AD brain tissue^14–16^, which efficiently seeds tau pathology^16^ and induces memory impairment in mice^15^. Microglial EV secretion has been shown to be critical for the spread of pathological tau, and depleting microglia or inhibiting EV synthesis reduces tau propagation in tauopathy mouse models^17–19^. Thus, targeting molecules that drive microglial EV secretion represents a promising strategy to halt tau pathology progression.

P2X purinergic receptor 7 (P2RX7) is a promising target because of its inflammatory role and high expression in glia in AD and other tauopathy brains^20–23^. P2RX7, an ATP-gated Na^+^/Ca^2+^ channel, is highly expressed in microglia surrounding Aβ plaques^20, 21^. P2RX7 is activated by high eATP levels, promoting membrane depolarization, inflammasome activation, and secretion of proinflammatory cytokines and EVs^24–28^. In tauopathy mouse models, previous studies have suggested that P2RX7 inhibition or deletion improves cognition and reduces tau accumulation^22, 29, 30^. Our previous study revealed that treatment with GSK1482160, a CNS-penetrant P2RX7 antagonist, reduces ATP-induced secretion of EVs containing tau from microglia and misfolded tau accumulation, restoring cognitive function in a young PS19 tauopathy mouse model^29^. *P2rx7* deletion also disrupts EV secretion from microglia and astrocytes^28^. However, the exact mechanism by which P2RX7 contributes to the acceleration of tauopathy, neuroinflammation and neurodegeneration remains poorly understood.

We hypothesized that P2RX7 drives tau pathology via microglial EV secretion and inflammatory activation. To test this hypothesis, we used genetic and cell type-specific disruption of *P2rx7* in mouse brains and conducted multiomics-based comprehensive analysis of brain tissues and brain-derived EVs (BDEVs) from PS19 tauopathy mice. We demonstrated that *P2rx7* deficiency preserved cognition, reduced neurodegeneration, and prevented microglial transition to an inflammatory phenotype associated with increased EV production in PS19 mice. *P2rx7* deficiency also suppressed microglial EV secretion, and cell type-specific targeting of *P2rx7* in microglia suppressed tau propagation. We detected a P2RX7-dependent increase in the number of EVs containing tau and mitochondrial molecules, which triggered a microglial inflammatory response in tauopathy model mice after intracranial injection *in vivo*. Here, we propose that P2RX7 is a critical regulator of EV-mediated spread of tau pathology and neuroinflammation, underscoring its therapeutic potential in AD.

## Results

### P2RX7 deletion protects against cognitive impairment and neurodegeneration in PS19 tau transgenic mice

**Figure 1:**
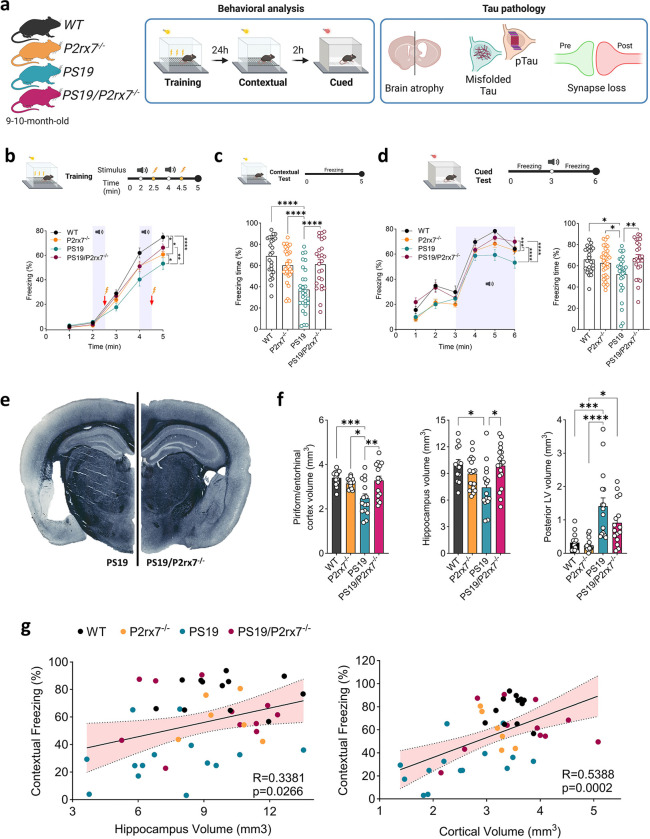
*P2rx7* deficiency protects against cognitive impairment and neurodegeneration in PS19 tau transgenic mice. **a,** Schematic design for evaluating the effects of P2rx7 on tau pathology in 9–10-month-old PS19 mice. **b-d**, Effects of P2rx7 genetic ablation on memory impairment in PS19 mice in the contextual (**c**) and cued (**d**) fear conditioning tests (*n=* 23 WT mice, *n=* 30 P2rx7^−/−^ mice, *n=* 28 PS19 mice, *n=* 25 PS19/P2rx7^−/−^ mice; one-way ANOVA with Holm–Šidák post hoc analysis). **e**, Representative images of PS19 and PS19/P2rx7^−/−^ mouse brain sections stained with Sudan black. **f-h**, Volumetric analysis of piriform/entorhinal cortex (*n=* 14 WT mice, *n*= 15 P2rx7^−/−^ mice, *n=* 15 PS19 mice, *n=* 15 PS19/P2rx7^−/−^ mice), hippocampus (*n=* 14 WT mice, *n*= 18 P2rx7^−/−^ mice, *n=* 15 PS19 mice, *n=* 19 PS19/P2rx7^−/−^ mice), and posterior lateral ventricle (LV) (*n=* 13 WT mice, *n*= 16 P2rx7^−/−^ mice, *n=* 16 PS19 mice, *n=* 16 PS19/P2rx7^−/−^ mice); one-way ANOVA with Holm–Šidák post hoc analysis. **g**, Correlations between contextual freezing memory and hippocampal and cortical (piriform/entorhinal cortex) volumes (*n=* 43; Pearson correlation analysis). All bar graphs represent the mean ± SEM; *P < 0.05, **P < 0.01 and ****P < 0.0001.

To investigate the role of *P2rx7* in the development of tau pathology, we crossed *P2rx7* knockout (P2rx7^−/−^) mice with a PS19 tauopathy mouse model, which overexpresses P301S mutant human tau (Fig. 1a). Previous reports have suggested impairment in the contextual fear conditioning test (FC) in PS19 mice^29, 31^. We thus assessed associative learning and memory function in 9–10-month-old mice via FC and observed significant deficits in fear memory acquisition as well as contextual and cued memory in PS19 mice compared with wild-type (WT) controls, which is indicative of hippocampal and amygdala dysfunction^31^ (Fig. 1b). In contrast, PS19/P2rx7^−/−^ mice presented preserved memory acquisition and function in both contextual and cued tests compared to PS19 mice (Fig. 1c, d). Given that *P2rx7* deficiency preserved learning and memory function in PS19 mice, we examined the severity of neurodegeneration in PS19 and PS19/P2rx7^−/−^ mice. Compared with WT mice, PS19 mice presented pronounced brain atrophy, characterized by a reduced volume in the piriform/entorhinal cortex and hippocampus and enlargement of the posterior lateral ventricle (LV). However, *P2rx7* deficiency significantly preserved cortical and hippocampal volume and reduced the enlargement of the ventricles in PS19/P2rx7^−/−^ mice (Fig. 1e, f). Importantly, hippocampal and cortical volumes were significantly positively correlated with contextual freezing behavior (Fig. 1g), suggesting that *P2rx7* deficiency synergistically ameliorated neurodegeneration and preserved memory function in PS19 mice.

### P2rx7 deficiency attenuates tau pathology and synaptic loss in PS19 mice

**Figure 2:**
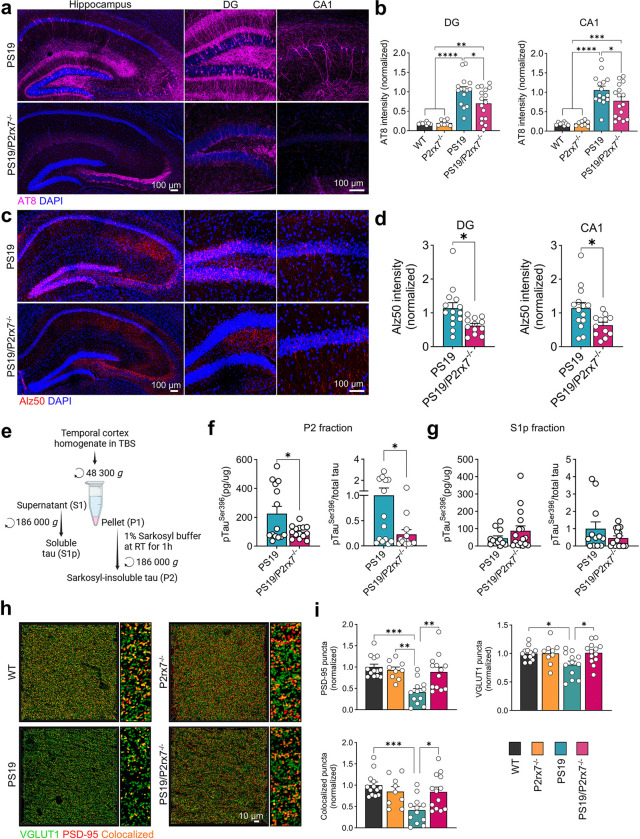
*P2rx7* deficiency attenuates tau pathology in PS19 mice. **a-d**, Representative images and quantification of phosphorylated tau (AT8, **a, b**) (*n=* 8 WT mice, *n=* 8 P2rx7^−/−^ mice, *n=* 15 PS19 mice, *n=* 16 PS19/P2rx7^−/−^ mice; one-way ANOVA with Holm–Šidák post hoc analysis) and misfolded tau (Alz50, **c, d**) (*n=* 14 PS19 mice, *n=* 12 PS19/P2rx7^−/−^ mice; two-tailed Student’s t test) in the DG and CA1 hippocampus areas of 9–10-month-old mice. **e-g**, ELISA measurement of human pTau-Ser396 in sarkosyl-insoluble (P2) (*n=* 12 PS19 mice, *n=*16 PS19/P2rx7^−/−^ mice) and soluble (S1p) (*n=* 14 PS19 mice, *n=* 14 PS19/P2rx7^−/−^ mice) fractions isolated from the temporal cortex of PS19 and PS19/P2rx7^−/−^ mice; two-tailed Student’s t test. **h**, Representative confocal images and quantification of the hippocampal CA1 Vglut1 and PSD95 colocalized synaptic puncta after 3D rendering using Imaris (*n=* 13 WT mice, *n=* 9 P2rx7^−/−^ mice, *n=* 12 PS19 mice, *n=* 13 PS19/P2rx7^−/−^ mice; one-way ANOVA with Holm–Šidák post hoc analysis). All bar graphs represent the mean ± SEM; *P < 0.05, **P < 0.01, ***P < 0.001 and ****P < 0.0001. DG: Dentate gyrus, CA1: Cornu ammonis 1.

We next evaluated the effects of *P2rx7* deficiency on tau pathology in PS19 mice. Compared with WT mice, PS19 mice exhibited robust phosphorylated tau, as evidenced by increased AT8^+^ pTau^Ser202/Thr205^ intensity in the hippocampal dentate gyrus (DG) and cornu ammonis 1 (CA1) region (Fig. 2a, b). PS19/P2rx7^−/−^ mice showed significantly lower AT8^+^ intensity (Fig. 2a, b) and reduced Alz50^+^ or MC1^+^ misfolded tau accumulation in the hippocampus (Fig. 2c, d for Alz50 and Extended Data Fig. 1a for MC1). In addition, sequential fractionation of temporal cortical brain lysates revealed significantly lower pTau^Ser396^ levels in the sarkosyl-insoluble P2 fractions from PS19/P2rx7^−/−^ mice than in those from PS19 mice, confirming the decreased accumulation of fibrillar tau in PS19 mice caused by *P2rx7* deficiency (Fig. 2e–g).

As tau pathology is often associated with synaptic loss^32–34^, we assessed synaptic integrity by quantifying vesicular glutamate transporter 1 (VGLUT1, a presynaptic marker) and postsynaptic density 95 (PSD-95, a postsynaptic marker) puncta in the CA1 region. *P2rx7* deficiency significantly preserved all the synaptic marker^+^ puncta in PS19 mice (Fig. 2h, i). Overall, these results demonstrate that *P2rx7* deficiency efficiently reduces tau aggregation and accumulation and protects synaptic integrity and brain parenchymal volume in PS19 mice.

### Conditional knockout of P2rx7 in microglia abolishes tau propagation from the entorhinal cortex to hippocampus

**Figure 3:**
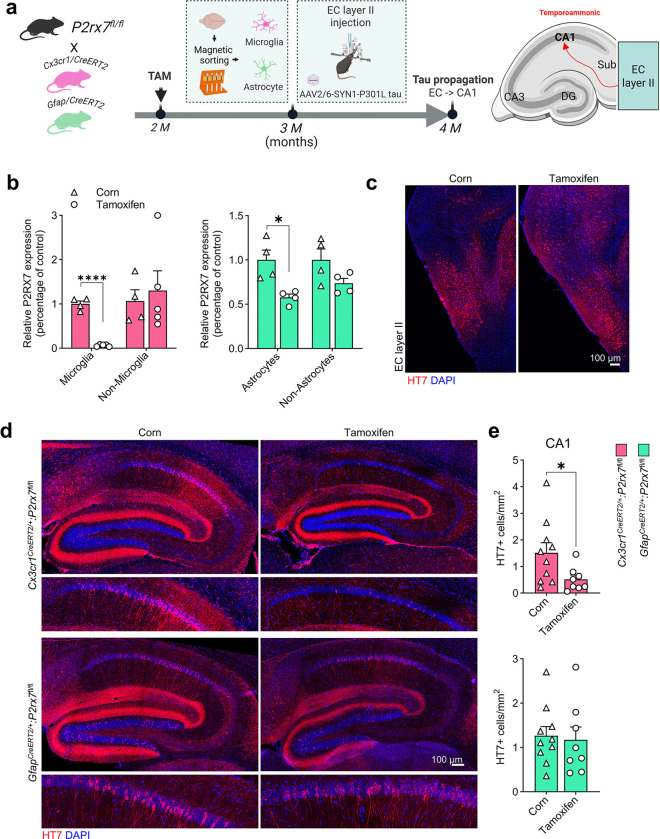
Cell type-specific deletion of *P2rx7* in microglia halts tau propagation from the entorhinal cortex to the hippocampal CA1 region. **a**, Schematic design for evaluating the effect of *P2rx7* conditional knockout (cKO) on tau propagation in the mouse brain. *P2rx7*^fl/fl^ mice were crossed with *Cx3cr1*^*CreERT2*^ and *Gfap*^*CreERT2*^ mice, and tamoxifen induction of Cre recombinase at 2 months of age. At 3 months of age, mice were injected with AAV2/6-SYN1-P301L tau in the entorhinal cortex (EC) layer II for mutant human tau (Tau^P301L^) expression in neurons. After 1 month of incubation, human tau propagation to the hippocampal CA1 region was assessed. **b**, Conditional knockout validation of *P2rx7* expression levels by RT–qPCR in microglia and astrocytes isolated from mice incubated for 1 month after tamoxifen injection (*n=* 4 *Cx3cr1-CreERT2* + Corn mice, *n=* 5 *Cx3cr1-CreERT2* + Tamoxifen mice, *n=* 4 *Gfap-CreERT2* + Corn mice, *n=* 4 *Gfap-CreERT2* + Tamoxifen mice; two-tailed Student’s t test). **c**, Representative confocal images of human tau (HT7) staining in EC from 4-month-old mice injected with corn oil or tamoxifen. **d, e**, Representative confocal images of mouse hippocampi (d) and quantification of the number of HT7+ microglia and astrocytes in the hippocampal CA1 region of P2rx7cKO mice (e). The graphs present the total number of HT7+ cells in CA1 normalized by the HT7 intensity in the injected EC region (*n=* 10 *Cx3cr1-CreERT2* + Corn mice, *n=* 8 *Cx3cr1-CreERT2* + Tamoxifen mice, *n=* 10 *Gfap-CreERT2* + Corn mice, *n=* 8 *Gfap-CreERT2* + Tamoxifen mice; two-tailed Student’s t test). All bar graphs represent the mean ± SEM; *P < 0.05 and ****P < 0.0001. CA1: cornu ammonis 1.

To address the cell type-specific contribution of *P2rx7* to tau propagation *in vivo*, we generated a conditional knockout (cKO) mouse model for microglial and astrocytic *P2rx7* deletion by crossing *P2rx7*^fl/fl^ mice with *Cx3cr1-CreERT2*^35^ and *GFAP-CreERT2*^36^ mice, respectively (*Cx3cr1*^*CreERT2*^:*P2rx7*^fl/fl^ and *GFAP*^*CreERT2*^:*P2rx7*^fl/fl^ mice). We used our tau propagation mouse model, in which tau propagates from neurons in the entorhinal cortex (EC) to the CA1 region of the hippocampus via the temporoammonic pathway^37^, which is achieved by stereotaxic injection of AAV2/6-SYN1-P301L tau into layer II of the EC one month after Cre recombinase-mediated *P2rx7* deletion by tamoxifen injection (Fig. 3a). We confirmed the knockdown efficiency of *P2rx7* in isolated CD11b^+^ microglia (99.9 ± 0.02%) and ACSA2^+^ astrocytes (57.7 ± 0.07%) after tamoxifen injection (Fig. 3b). Immunostaining of mouse brain tissue sections confirmed the expression of HT7^+^ human tau in the EC region (Fig. 3c) and its significantly reduced propagation to the CA1 region in microglia-specific *P2rx7* cKO mice (*Cx3cr1*^*CreERT2*^:*P2rx7*^fl/fl^ with tamoxifen injection, Fig. 3d, e) but not in astrocyte-specific *P2rx7* cKO mice (*GFAP*^*CreERT2*^:*P2rx7*^fl/fl^ with tamoxifen injection, Fig. 3d, e) compared with corn oil-injected groups. These findings indicate that microglial *P2rx7* plays a critical role in tau propagation.

### P2rx7 induces inflammatory glial activation and enriched EV signatures in the hippocampal tissues of PS19 mice

**Figure 4:**
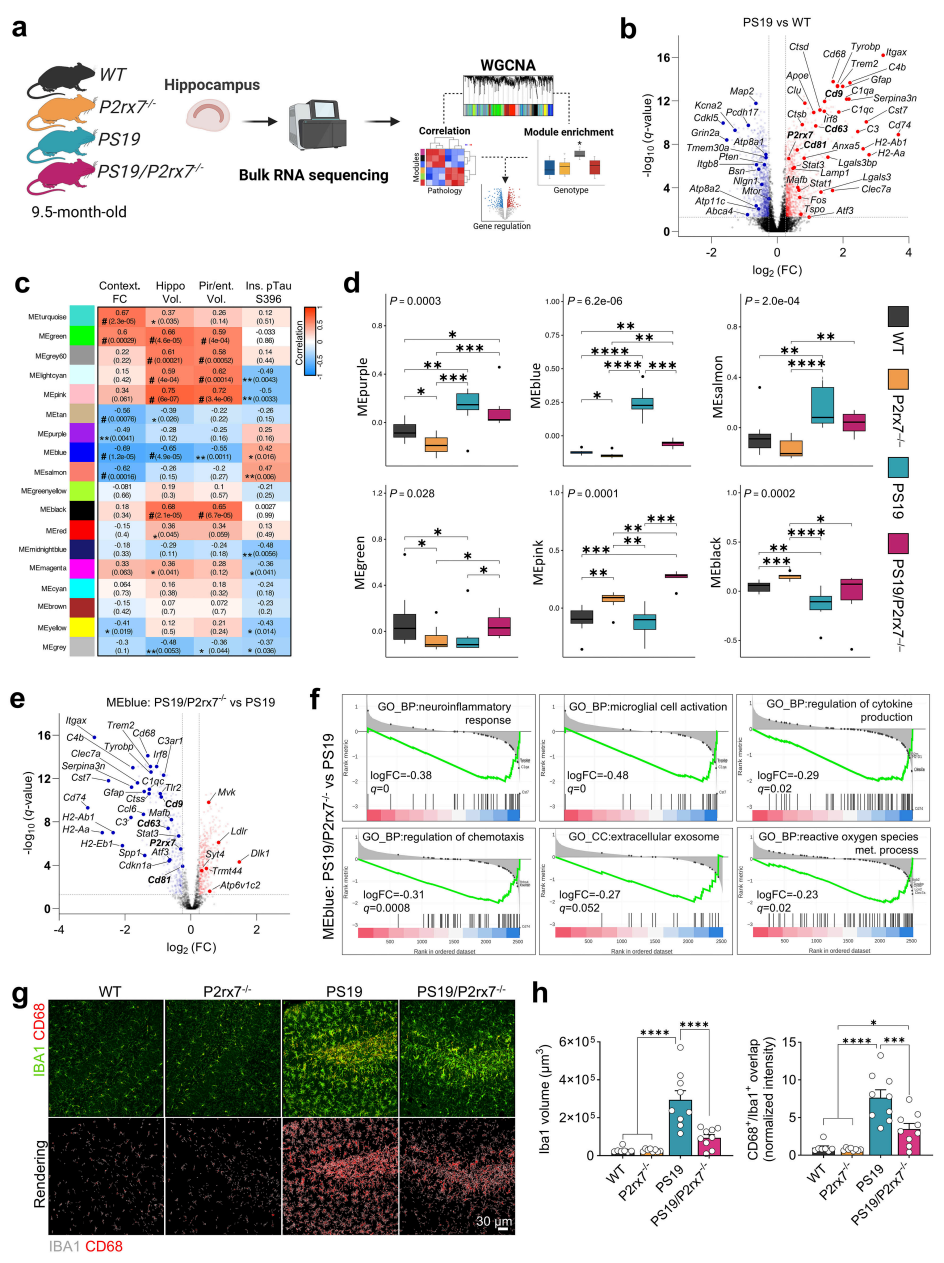
P2rx7 deficiency ameliorates inflammatory microglial activation in PS19 mice. **a,** Schematic design for bulk RNA-seq analysis of hippocampi from 9.5-month-old mice (*n=* 8 WT mice, *n=* 8 P2rx7^−/−^ mice, *n=* 10 PS19 mice and *n=* 6 PS19/P2rx7^−/−^ mice). **b**, Volcano plot showing DEGs between PS19 and WT mice (*q-*value < 0.05). **c**, Weighted gene co-expression network analysis (WGCNA) module correlations with key pathological phenotypes from PS19 mice (*n=* 8 WT mice, *n=* 8 P2rx7^−/−^ mice, *n=* 10 PS19 mice and *n=* 6 PS19/P2rx7^−/−^ mice). **d**, Comparison of the first principal component of each module (eigengene) per genotype using the top 6 significantly correlated modules with at least one phenotype (*n=* 8 WT mice, *n=* 8 P2rx7^−/−^ mice, *n=* 10 PS19 mice and *n=* 6 PS19/P2rx7^−/−^ mice; Kruskal–Wallis test with Wilcoxon test post hoc analysis). **e**, Volcano plot showing DEGs between PS19/P2rx7^−/−^ and PS19 mice in the MEblue module (*n=* 10 PS19 mice, *n=* 6 PS19/P2rx7^−/−^ mice; *q-*value < 0.05). **f**, Gene set enrichment analysis (GSEA) of the top pathways regulated in PS19/P2rx7^−/−^ versus PS19 mice in the MEblue module (*n=* 10 PS19 mice, *n=* 6 PS19/P2rx7^−/−^ mice). **g**, Representative confocal images and 3D rendering of microglial IBA1 and CD68 staining in the DG hippocampus. **h**, IBA1 volume and CD68/IBA1 colocalized volume were measured using Imaris (*n=* 7 WT mice, *n=* 8 P2rx7^−/−^ mice, *n=* 9 PS19 mice, *n=* 9 PS19/P2rx7^−/−^ mice; one-way ANOVA with Holm–Šidák post hoc analysis). All bar graphs represent the mean ± SEM; *P < 0.05, **P < 0.01, ***P < 0.001 and ****P < 0.0001.

To investigate the transcriptional changes driven by *P2rx7* deficiency in PS19 mice, we performed bulk RNA-seq of hippocampal tissues at 9.5 months of age (Fig. 4a). PS19 mice presented increased expression of differentially expressed genes (DEGs) associated with disease-associated and neurodegenerative microglia (DAM/MGnD), including *Cd68, Clec7a, Apoe,* and *Itgax*^38, 39^, as well as the reactive astrocytic gene (*Gfap*), disease-associated astrocyte (DAA)^40^ and oligodendrocyte (DAO)^41^ genes, such as *C4b* and *Serpina3n* (Fig. 4b and Supplementary Table 1). *P2rx7* expression was also increased in PS19 mice (*q*-value= 6.70) (Fig. 4b) and associated with specific upregulation or alternative splicing of exon 13, the region deleted in P2rx7^−/−^ mice (Extended Data Fig. 1b-d), producing a truncated, nonfunctional transcript^42^. Gene set enrichment analysis (GSEA) revealed increased inflammation, adaptative immune response, type II interferon signaling, microglial activation, extracellular exosome, and oxidative phosphorylation pathways in PS19 mice compared with WT mice, alongside the downregulation of learning, memory and excitatory/inhibitory synapses (Extended Data Fig. 2a). *P2rx7* deficiency in PS19 mice significantly suppressed several inflammatory pathways, including inflammasome activation, cytokine production, type II interferon responses, and microglial activation, and downregulated the extracellular exosome and reactive oxygen species (ROS) metabolism pathways (Extended Data Fig. 2b).

We applied weighted gene co-expression network analysis (WGCNA)^43^ to identify key gene modules associated with disease phenotypes—contextual memory, brain volume, and insoluble pTau^S396^ level (Supplementary Table 1). Using the eigengene (the first principal component of a given module), we identified six modules that were significantly correlated with at least one phenotype and inversely regulated in PS19/P2rx7^−/−^mice compared with PS19 mice (Fig. 4c, d). MEgreen, MEpink, and MEblack represent neuroprotective modules that were upregulated in PS19/P2rx7^−/−^ and positively correlated with increased brain volume, whereas MEpurple, MEblue, and MEsalmon were negatively correlated with FC performance, suggesting their pathogenic roles (Fig. 4c, d). Notably, MEblue was strongly correlated with all disease traits and represented a gene signature unique to PS19 mice (Fig. 4c, d and Extended Data Fig. 2c).

Analysis of DEGs from MEblue module revealed that *P2rx7* deficiency significantly downregulated DAM/MGnD markers (e.g., *Clec7a, Cd9,* and *Itgax*) and DAA/DAO genes (e.g., *C4b* and *Serpina3n*) in the hippocampus (Fig. 4e). GSEA of MEblue indicated the suppression of pathways linked to neuroinflammation, cytokine production, microglial activation, chemotaxis, ROS generation, and extracellular exosomes (Fig. 4f). Immunostaining confirmed that microglial activation was reduced, with decreased Iba1^+^ microglial volume and CD68^+^ phagocytic cells in the hippocampus of PS19/P2rx7^−/−^ mice compared with those in PS19 mice (Fig. 4g, h). Importantly, beyond glial activation, the regulation of exosome/EV-related genes (*Cd9, Cd81, and Cd63*) within MEblue further highlights the role of EVs in tau pathology (Fig. 4f and Extended Data Fig. 2d).

These findings emphasize the critical role of P2RX7 in modulating glial activation and EV secretion, underscoring its importance as a key regulator of neuroinflammation and tau-associated pathology in PS19 mice.

### P2rx7 is highly expressed in microglia and regulates the oxidative stress response in glial cells in PS19 mice

To investigate the molecular mechanisms of P2RX7 signaling in specific cell types involved in tauopathy, we performed scRNA-seq on brain tissues from 9.5-month-old WT, PS19, and PS19/P2rx7^−/−^ mice via the sequential barcoding platform from Parse Biosciences (Extended Data Fig. 3a). After stringent quality control, 89,795 high-quality single cells were obtained and annotated into distinct clusters representing major CNS cell types (Extended Data Fig. 3b-e). Notably, *P2rx7* deficiency differentially regulates genes across all identified cell types in PS19 mice (Extended Data Fig. 3f and Supplementary Tables 2 and 3). Cell type composition analysis revealed increased population of microglia and astrocytes and reduced population of oligodendrocytes in PS19 mice, which were reversed in PS19/P2rx7^−/−^ mice. Only two minor immature and excitatory neuronal clusters were identified in our dataset (Extended Data Fig. 4a, b).

According to the scRNA-seq data, *P2rx7* was highly expressed in microglia and two other minor clusters of immune cells and oligodendrocyte progenitor cells (OPCs) (Extended Data Fig. 4c). We next examined genes commonly regulated in glia and identified 242 common DEGs between PS19 and PS19/P2rx7^−/−^ mice (Extended Data Fig. 4d and Supplementary Table 4). These genes were associated mainly with cellular detoxification and oxidative stress, indicative of a protective response to oxidative stress and senescence (Extended Data Fig. 4e). Network analysis of the common genes revealed the downregulation of networks encoding selenoproteins with antioxidant functions (*Selenop, Selenos, and Selenok*), protein folding/endoplasmic reticulum stress (*Hspa8, Dnajb9, Manf,* and *Xbp1*), autophagy/mitophagy genes (*Ubb, Ubc, Map1lc3b, and Gabarapl1*), the downregulation of *Tmem30a* and the upregulation of *Atp8a2* phospholipid flippase complex. Intriguingly, genes associated with mitochondrial complexes/oxidative phosphorylation (*Ndufa4, Ndufab11, Ndufs8, Cyc1, and Cycs*) were significantly downregulated in all PS19/P2rx7^−/−^ glia (Extended Data Fig. 4e, f and Supplementary Table 4).

Taken together, these results suggest that *P2rx7, which is* highly expressed in microglia, may regulate glial mitochondrial oxidative stress in PS19 mice and that *P2rx7* deficiency may reduce glial mitochondrial damage and cellular stress in the context of tauopathy.

### P2rx7 regulates microglial transition to an inflammatory and EV hypersecretory state in tauopathy

Given that microglia highly express the *P2rx7 gene*and expand their population under tauopathy, we investigated microglia-specific gene signatures under *P2rx7* deficiency in PS19 mice. Analysis of microglial DEGs from PS19 versus WT mice revealed downregulation of mitophagy genes (*Pink1, Tomm7, and Mfn2*) and *Atp8a2*. Microglia from PS19 mice presented increased expression of stress-activated genes (*Atf3, Jun, and Fos*) and chemokines (*Ccl2, Ccl3, and Ccl4*), with pathway analysis revealing the activation of apoptosis, senescence, and the NF-κB, TNF-α, and MAPK signaling pathways (Extended Data Fig. 4g, h and Supplementary Table 5).

**Figure 5:**
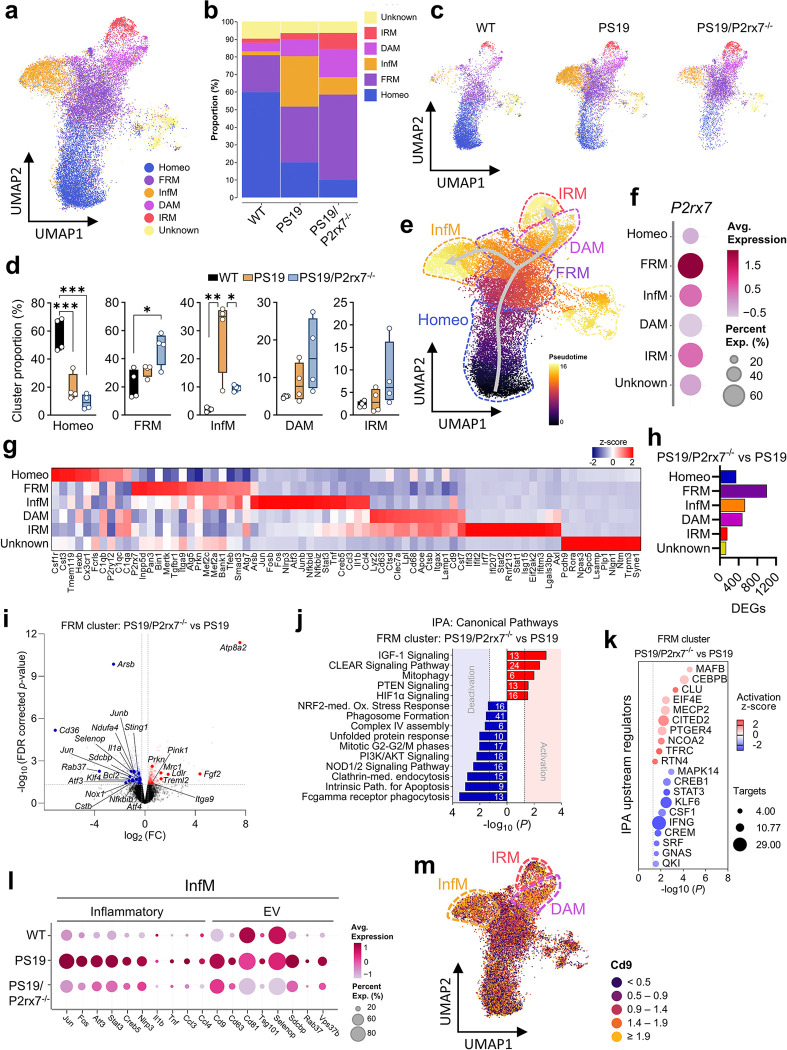
P2RX7 regulates tauopathy-induced conversion of microglia to an inflammatory phenotype. a, Uniform manifold approximation and projection (UMAP) plot representation of microglial subclusters. **b**, Stacked bar plot showing the subcluster compositions of microglia in WT, PS19 and PS19/P2rx7^−/−^. **c**, UMAP representation of microglial subclusters across genotypes. Subcluster annotation is indicated by the colors shown in **b**. **d**, Box and whisker plot comparing microglial subclusters among genotypes. **e**, Phenotypic trajectory analysis of microglia obtained by unbiased pseudotime ordering using Monocle3. The dashed lines are colored according to each cluster, as shown in **a**. **f**, Normalized average expression showing the expression of *P2rx7* in microglial subclusters. **g**, Selection of the top DEGs and homeostatic genes enriched in each cluster. **h**, Number of DEGs regulated by *P2rx7* deficiency in each microglial cluster in PS19 mice. **i**, Volcano plot showing DEGs regulated in FRM cluster from PS19/P2rx7^−/−^ mice. **j**, Ingenuity pathway analysis (IPA) of canonical pathways in FRM cluster from PS19/P2rx7^−/−^ mice. **k**, IPA of the top predicted upstream regulators in FRM cluster from PS19/P2rx7^−/−^ mice. **l**, Normalized average expression of inflammatory and EV genes in InfM cluster. **m**, UMAP plot representation of *Cd9* expression in microglia clusters InfM (orange), DAM (magenta) and IRM (red). All bar graphs represent the mean ± SEM; *P < 0.05, **P < 0.01 and ***P < 0.001.

To determine how *P2rx7* disruption affects the microglial response to tauopathy, we performed subclustering analysis of microglia (Extended Data Fig. 5a, b). After filtering out immune cells, low-quality cells, and doublets (Extended Data Fig. 5c), microglia were subclustered into six distinct subpopulations, annotated according to previous literature^38, 39, 44, 45^ as homeostatic microglia (Homeo), inflammatory microglia (InfM) related to cytokine production, DAM/MGnD, interferon-responsive microglia (IRM), a novel first-responding microglia (FRM) with a phagocytic signature, and a smaller nonidentifiable cluster (Unknown) (Fig. 5a and Extended Data Fig. 5d-e). In WT mice, the Homeo cluster was dominant, whereas in PS19 mice it presented transitions to the InfM, FRM, DAM, and IRM states (Fig. 5b). The InfM cluster expanded significantly and became one of the dominant clusters in PS19 mice compared with WT mice, whereas it was markedly reduced, with a corresponding expansion of FRM cluster in PS19/P2rx7^−/−^ mice (Fig. 5b–d).

We conducted phenotypic trajectory analysis and pseudotime ordering to track microglial conversion from homeostatic to disease-associated states. FRM was identified as a transitional state, moving toward differentially activated states such as InfM and DAM, with DAM transitioning into the IRM state (Fig. 5e). *P2rx7* was predominantly expressed in the FRM (Fig. 5f, g and Supplementary Table 6), confirming its critical role in the conversion of microglia from the FRM state to the InfM state, the transition of which was markedly reduced in PS19/P2rx7^−/−^ mice (Fig. 5b–d). Compared with all other microglial clusters, the FRM cluster also presented a distinct gene signature, with enrichment of key AD GWAS genes (*Inpp5d, Bin1, Mef2c*), microglial signaling genes (*Tgfbr1, Mertk, Smad3*), and autophagy/mitophagy genes (*Atg5, Atg7, Prkn*) (Fig. 5g). FRM also showed the greater number of DEGs regulated among microglial clusters in PS19/P2rx7^−/−^mice compared with PS19 mice (Fig. 5h).

Comparison of DEGs in FRM between PS19/P2rx7^−/−^and PS19 mice revealed upregulation of *Atp8a2, Pink1, Prkn, Ldlr,* and *Fgf2* and downregulation of inflammatory and stress-associated genes, including *Cd36, Sting1, Cstb, Bcl2, Atf3, Jun, Selenop,* and *Rab37* (Fig. 5i). Some of these genes, including *Atp8a2, Prkn, Cd36, Bcl2, Selenop,* and *Rab37,* were also similarly regulated in InfM between PS19/P2rx7^−/−^and PS19 mice (Extended Data Fig. 5f and Supplementary Table 7), indicating that changes in FRM may ultimately affect the InfM phenotype. Ingenuity pathway analysis revealed that FRM in PS19/P2rx7^−/−^ mice activated canonical pathways, including the IGF-1, PTEN and CLEAR signaling, and mitophagy but downregulated the phagocytosis, apoptosis, endocytosis and unfolded protein response pathways (Fig. 5j). This shift led to reduced cell movement, decreased inflammatory responses and decreased cell trafficking in PS19/P2rx7^−/−^ FRM microglia (Extended Data Fig. 5g), in which IFNγ, STAT3, CREB1, and CREM are predicted to be suppressed upstream regulators (Fig. 5k and Extended Data Fig. 5h). These findings align with the reduced interferon II (IFNγ) response (Extended Data Fig. 2b) and *Stat3* expression in the PS19/P2rx7^−/−^ mousehippocampus (Fig. 4f) and PS19/P2rx7^−/−^ InfM microglia (Fig. 5l), suggesting that P2rx7-mediated IFNγ and STAT3 signaling may be required for microglial conversion to a proinflammatory state in tauopathy and that *P2rx7* disruption reduces neuroinflammation.

To explore whether *P2rx7* disruption reduces microglial EV secretion in tauopathy, we analyzed EV-related gene expression in microglial clusters. We observed elevated expression of EV markers, such as *Cd9*, in InfM, DAM, and IRM (Extended Data Fig. 5i). DAM and IRM also upregulated other EV markers, *Cd63* and *Cd81*, whereas InfM showed increased expression of *Sdcbp*, *Vps37b*, and *Rab37*, which are linked to EV biogenesis and secretion^46–49^ (Extended Data Fig. 5i). Notably, the expression of these EV markers, including *Selenop*, a regulator of EV synthesis and secretion from activated microglia, was downregulated in PS19/P2rx7^−/−^ InfM microglia^50, 51^ (Fig. 5l). These data show enhanced EV biogenesis and secretion in PS19 InfM, DAM, and IRM microglia, which are dampened by *P2rx7* deletion.

### P2rx7 deficiency reduces EV secretion from tau-activated microglia in vivo

**Figure 6:**
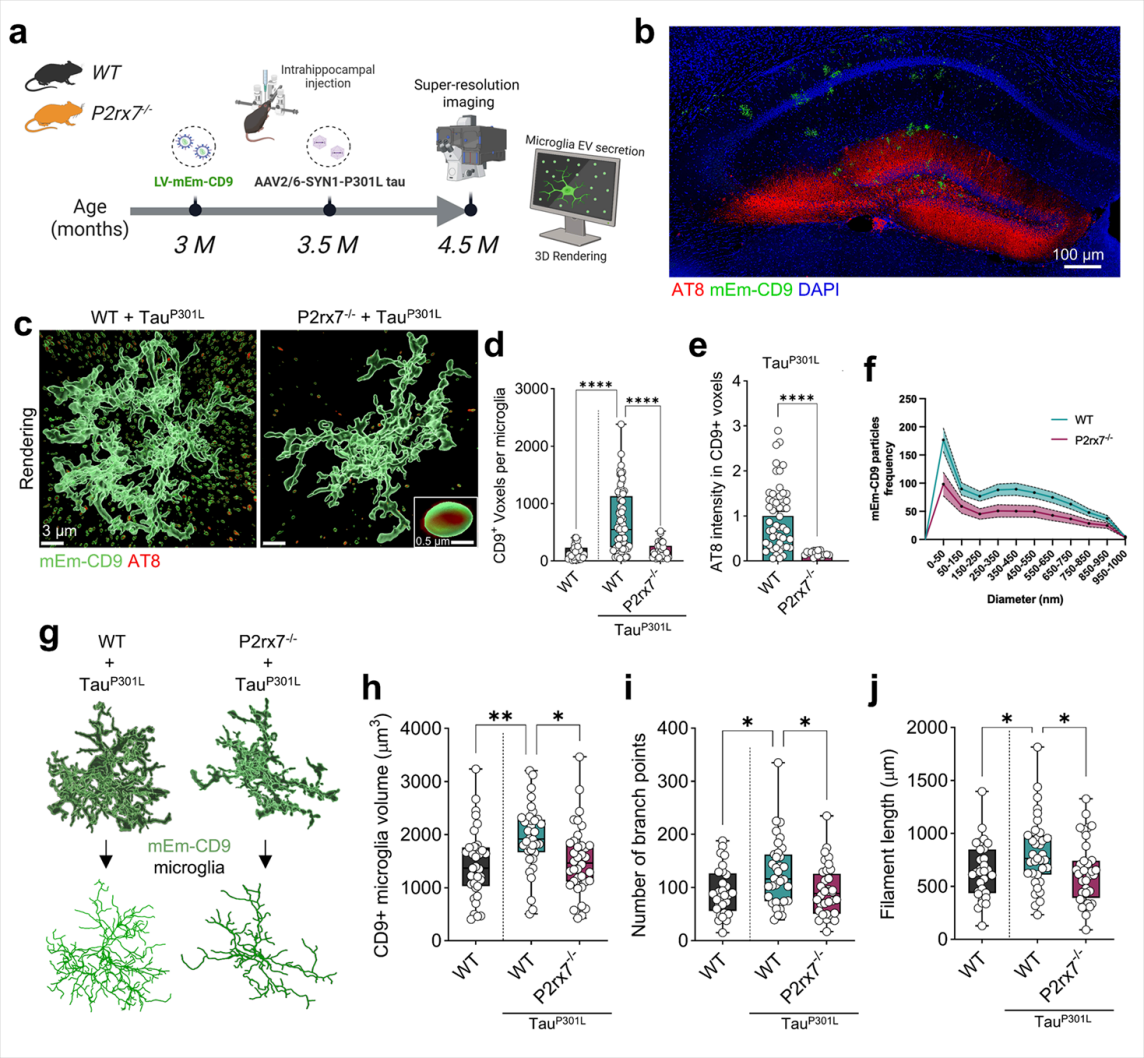
*P2rx7* deficiency suppresses EV secretion from activated microglia induced by tau pathology *in vivo*. **a**, Experimental scheme for lentivirus expression of mEmerald-CD9 in microglia and AAV2/6-SYN1-P301L tau expression of P301L tau (Tau^P301L^) in neurons in the hippocampal DG region from WT and P2rx7^−/−^ mice. **b**, Representative images of mEm-CD9^+^ microglia and p-Tau (AT8) in the hippocampi of injected mice. **c-f**, *In situ* analysis of mEm-CD9^+^/AT8^+^ microglial EV secretion and estimated EV size distribution rendered by Imaris (*n=* 6–9 microglia per mouse; *n=* 4 WT mice, *n=* 4 WT-Tau^P301L^ mice, *n=* 5 P2rx7^−/−^-Tau^P301L^; one-way ANOVA with Holm–Šidák post hoc analysis). **g-j**, Volume, ramification and filament analysis of mEm-CD9^+^ microglia from WT and P2rx7^−/−^ mice expressing Tau^P301L^ (*n=* 6–9 microglia per mouse, *n=* 4 WT mice, *n=* 4 WT-Tau^P301L^ mice, *n=* 5 P2rx7^−/−^-Tau^P301L^; one-way ANOVA with Holm–Šidák post hoc analysis). All bar graphs represent the mean ± SEM; *P < 0.05, **P < 0.01 and ****P < 0.0001.

*Cd9*, a common EV marker, was found across all microglial disease states (Fig. 5m and Extended Data Fig. 5i). We previously tracked microglial EV secretion *in vivo*^51^ by employing a lentivirus expressing CD9 fused with mEmerald (mEm-CD9)^18, 51^in a microglia-specific manner via the tandem sequence of miR9 at the 3’ UTR. To determine whether *P2rx7* deficiency may changemicroglial function to secrete EVs, we coinjected mEm-CD9 lentivirus and AAV2/6-SYN1-P301L tau-expressing Tau^P301L^ in neurons under the synapsin-1 promoter in 3-month-old WT and *P2rx7*^−/−^ mice and measured the number of GFP^+^/CD9^+^ particles *in vivo* at 4 weeks post injection (Fig. 6a, b). Super-resolution confocal imaging of the hippocampal region revealed that WT mice expressing TauP301L showed a significant increase in mEm-CD9^+^ particles, which was significantly reduced in *P2rx7*^−/−^mice (Fig. 6c, d). This reduction in mEm-CD9^+^ particles was accompanied by markedly reduced AT8 intensity in mEm-CD9^+^ particles in *P2rx7*^−/−^mice (Fig. 6e). Furthermore, the number of mEm-CD9^+^ particles was lower in *P2rx7*^−/−^ mice expressing TauP301L than in WT mice across all sizes of EVs (Fig. 6f). In addition, morphological assessment of mEm-CD9^+^ microglia revealed increased cell volume, branching, and process length of microglia in WT mice after Tau^P301L^ expression; these significant differences were diminished in *P2rx7*^−/−^mice(Fig. 6g–j), suggesting that tau pathology induces hypersecretion of EVs through microglial ramified processes. These results experimentally confirmed that tau-induced microglial activation drives hypersecretion of EVs, a change that is suppressed in *P2rx7*^−/−^mice. These findings indicate that *P2rx7* plays a critical role in EV-associated tau secretion, as observed in mEm-CD9^+^ microglia in Tau^P301L^-injected mice, thereby contributing to EV-mediated tau propagation.

### P2rx7 regulates the secretion of EV-associated tau and mitochondrial proteins correlated with disease progression in PS19 mice

**Figure 7:**
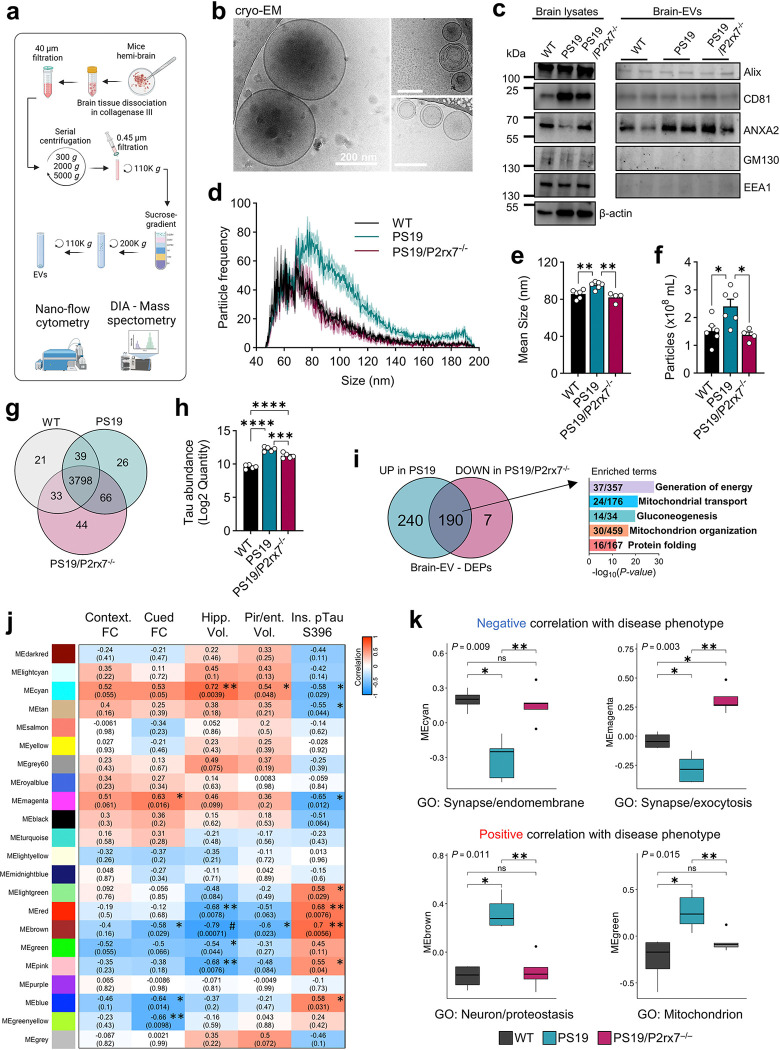
P2rx7 deficiency dampens pathological EV secretion and EV-protein network associated with disease phenotypes in PS19 mice. **a**, Schematic design for BDEV isolation from mice for nanoflow cytometry analysis and data-independent acquisition (DIA) mass spectrometry. **b**, Representative cryo-EM images of BDEVs isolated from the brains of 9.5-month-old mice. **c**, Western blot analysis of the brain and BDEV lysates from WT, PS19 and PS19/P2rx7^−/−^ mice for common EV (Alix, CD81 and ANXA2) and non-EV (GM130 and EEA1) protein markers. **d-f**, Nanoflow cytometry analysis of BDEV size distribution (**d** and **e**) and particle concentration (**f**) (*n=* 6 WT mice, *n=* 6 PS19 mice, *n=4* PS19/P2rx7^−/−^ mice; one-way ANOVA with Holm–Šidák post hoc analysis). **g**, Venn diagram displaying the number of BDEV proteins found per group (*n=* 5 mice per group). **h**, Tau protein abundance in BDEVs quantified by mass spectrometry analysis. **i**, Venn diagram and enriched pathway terms of differentially expressed proteins recovered by *P2rx7* deficiency in PS19 mice. **j,** BDEV protein module correlations with the main disease phenotypes. **k**, Comparison of the first principal component of each module (eigenprotein) by genotype using the top 4 significantly correlated modules (*n=* 5 WT mice, *n=* 5 PS19 mice and *n=* 5 PS19/P2rx7^−/−^ mice; Kruskal–Wallis test with Wilcoxon test post hoc analysis). Gene Ontology (GO) annotation was performed with the main DEPs from each module. All bar graphs represent the mean ± SEM; *P < 0.05, **P < 0.01, ***P < 0.001 and **** or ^#^ *P <* 0.0001.

EVs play a critical role in cell communication under both homeostatic and disease conditions. We previously reported elevated Aβ and tau contents in BDEVs isolated from postmortem human AD brain tissue^14–16^, further showing that these BDEVs can promote pathological tau seeding and memory impairment in mice^15, 16^, implicating EVs in tau propagation in AD. To investigate how P2rx7 modulates EV secretion in tauopathy, we isolated BDEVs from WT, PS19, and PS19/P2rx7^−/−^ mice using our established discontinuous sucrose gradient ultracentrifugation method (Fig. 7a)^52^. Cryogenic electron microscopy (cryo-EM) of BDEVs revealed preserved native morphology and structural heterogeneity, with a mean size of 150.9 nm (Fig. 7b and Extended Data Fig. 6a, b). BDEVs exhibit variations in the number of membrane layers and electron density, mostly electron-lucent EVs (79.4–86.1%) and some electron-dense EVs (13.9–20.6%) (Extended Data Fig. 6a, c). Immunoblotting of BDEVs confirmed the enrichment of common EV markers, including Alix, CD81 and ANXA2, whereas non-EV markers, including the Golgi apparatus marker GM130 and the endosomal protein EEA1, were depleted (Fig. 7c), confirming the enrichment of a purified EV fraction, as recommended by the Minimal Information for Studies of Extracellular Vesicles (MISEV2023)^53^. Nanoflow analyzer-based assessment of BDEVs at single-EV resolution revealed a significant increase in the mean size and particle count of BDEVs in PS19 compared with those in WT mice, which was normalized in PS19/P2rx7^−/−^ mice (Fig. 7d–f).

BDEVs samples were subjected to proteomic profiling using data-independent acquisition (DIA) mass spectrometry and identified a total of 4,027 proteins (Fig. 7g and Supplementary Table 8). Proteomic analysis revealed the presence of representative exosome and microvesicle markers in BDEVs (Extended Data Fig. 6d). Mouse BDEVs were highly enriched for representative cell type-specific EV markers, including neurons (ATP1A3, THY1, SNAP25 and NCAM1), oligodendrocytes (PLP1 and MOG), astrocytes (SLC1A2, SLC1A3, and GFAP), and microglia (ITGAM and P2RY12) (Extended Data Fig. 6e).

Comparison of differentially expressed proteins (DEPs) in BDEVs from PS19 and WT mice revealed 430 upregulated proteins, with tau (MAPT) showing predominant expression, and 282 downregulated proteins, including the microglial homeostatic marker P2RY12 (Extended Data Fig. 7a). Upregulated DEPs in BDEVs from PS19 mice were enriched in pathways related to mitochondrial functions, such as oxidative phosphorylation and mitochondrial protein degradation (Extended Data Fig. 7b). Notably, tau levels in BDEVs were markedly elevated in PS19 mice but significantly reduced in PS19/P2rx7^−/−^ mice (Fig. 7h), demonstrating the release of Tau^+^ EVs by P2RX7 in PS19 mouse brains.Strikingly, *P2rx7* deficiency reversed the upregulation of approximately 44% of the 430 DEPs (190 proteins) in PS19 BDEVs. These molecules were significantly enriched in mitochondrial and protein folding pathways (Fig. 7i and Extended Data Fig. 7c), suggesting that P2RX7 regulates the loading of mitochondrial proteins into BDEVs.

Our group previously applied WGCNA in proteomic datasets of human BDEVs as an unbiased tool to identify key molecular protein signatures correlated with pathological states in AD^54^. Here, we applied this method to classify BDEV protein co-expression modules and identified 10 modules, each showing at least one significant correlation with tau pathology-associated disease phenotypes in PS19 mice (Fig. 7j). We detected significant downregulation of MEcyan and MEmagenta—associated with synapses, endomembrane and exocytosis processes, as well as pathways such as mTOR signaling, and phagosome regulation—in the PS19 group, which were significantly restored in the PS19/P2rx7^−/−^group (Fig. 7k and Extended Data Fig. 7e, f). These modules were negatively correlated with insoluble pTau^S396^ levels and positively correlated with preserved brain volume (for MEcyan) or memory (for MEmagenta) (Fig. 7j) and overlapped with DEPs downregulated in the PS19 group (Extended Data Fig. 7d). In contrast, the MEbrown and MEgreen modules were significantly upregulated in the PS19 group and restored in the PS19/P2rx7^−/−^ group (Fig. 7k). Both were negatively correlated with brain volume, with MEbrown additionally showing a positive correlation with insoluble pTau^S396^ and a negative correlation with memory (Fig. 7j), overlapping with DEPs upregulated in the PS19 group (Extended Data Fig. 7d). MEbrown was enriched in neuron- and proteostasis-related proteins, including disease-associated proteins such as APOE and VCP (linked to AD and frontotemporal dementia), while MEgreen was enriched in mitochondrial proteins (e.g., NDUFA4, NDUFB11, NDUFS8, and CYC1) involved in the electron transport chain and oxidative phosphorylation (Extended Data Fig. 7e–g and Supplementary Table 8). Both modules were associated with neurodegenerative disease pathways, including AD (Extended Data Fig. 7e, g). These findings confirmed that BDEVs closely reflect the disease phenotypes of PS19 mice and their recovery by *P2rx7* deletion and carry mitochondrial molecules, which are strongly associated with the tauopathy phenotype, indicating that the mitochondrial content in EVs may play a role in propagating toxic molecules in the brain.

### P2RX7 regulates EV-mediated microglial activation via DNA-sensing and interferon signaling

**Figure 8:**
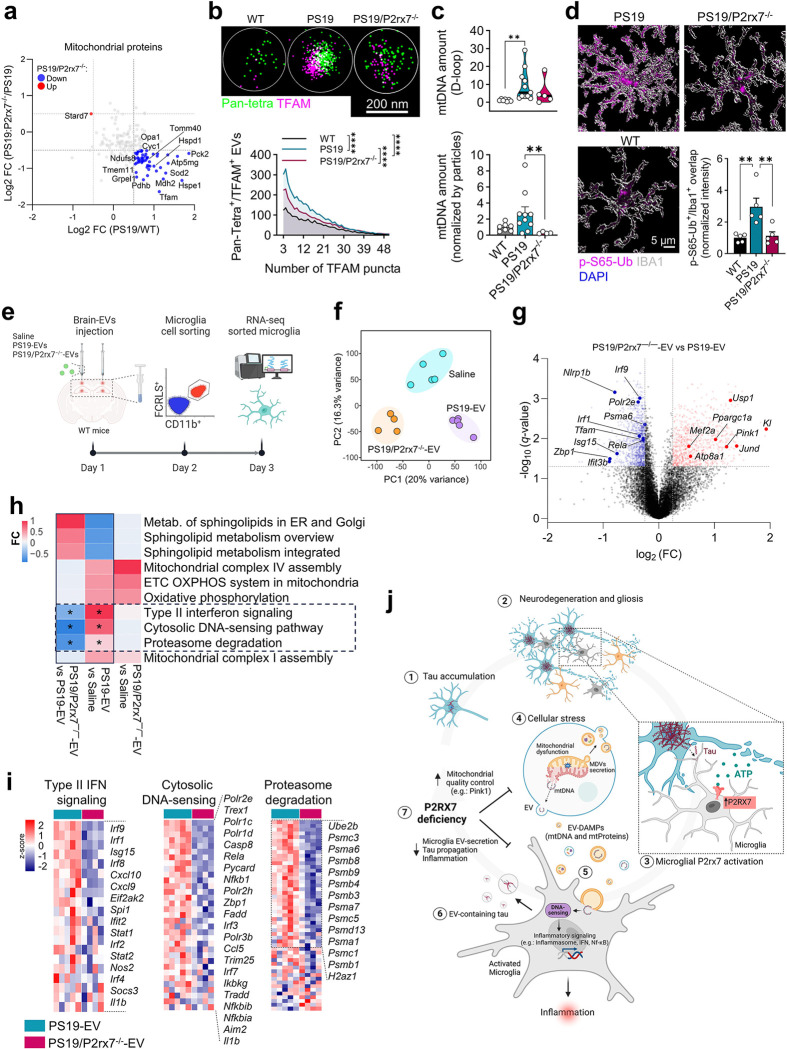
P2rx7 deficiency suppresses the activation of DNA-sensing/IFN signaling in microglia by reducing EV-mediated secretion of toxic mitochondrial molecules in mice with tauopathy. **a**, Volcano plot showing the regulation of mitochondrial proteins in BDEVs from PS19 and PS19/P2rx7^−/−^ mice (*n=* 5 mice per group). **b**, Single-molecule fluorescence analysis of TFAM on Pan-tetra^+^ EVs (CD63/CD81/CD9) was performed at the single-EV level via the CODI platform from ONI (*n*= 4 mice per group; two-way ANOVA with Holm–Šidák post hoc analysis). **c**, qPCR quantification of mtDNA (D-loop) in BDEVs normalized to WT-EVs (top) or EV particles (bottom) (*n=* 7 WT mice, *n=* 10 PS19 mice and *n=* 5 PS19/P2rx7^−/−^ mice; Kruskal–Wallis test with Wilcoxon test post hoc analysis). **d**, 3D rendering of p-S65-Ub/IBA1 colocalized intensity measurements in the DG hippocampus area using Imaris (*n=* 5 WT mice, *n=* 5 PS19 mice, *n=* 5 PS19/P2rx7^−/−^ mice; one-way ANOVA with Holm–Šidák post hoc analysis). **e**, Schematic of PS19, PS19/P2rx7^−/−^ BDEV or saline injection into the cortex and hippocampus of 9.5-month-old WT mice followed by microglia sorting and RNA-seq. **f**, Principal component analysis (PCA) of the transcriptome of microglia isolated from WT mice injected with BDEVs or saline (*n=* 5 saline, *n=* 5 PS19 BDEVs and *n=* 4 PS19/P2rx7^−/−^ BDEVs). **g**, Volcano plot showing DEGs regulated in microglia isolated from WT mice injected with PS19/P2rx7^−/−^ BDEVs versus those injected with PS19 BDEVs (*n=* 5 PS19 BDEVs and *n=* 4 PS19/P2rx7^−/−^ BDEVs, *q-*value < 0.05). **h**, Heatmap comparison of the top 10 pathways significantly regulated in microglia from WT mice injected with PS19 BDEVs (*P* < 0.05). **i**, Heatmap showing microglial regulation of the components of type II interferon (IFN) signaling, cytosolic DNA-sensing and proteasome degradation pathways by PS19-EVs and PS19/P2rx7^−/−^ BDEVs. **j**, Schematic summary of the contribution of the microglia-P2RX7-EV axis to tau pathology. The accumulation of phosphorylated tau aggregates leads to the disruption of neuronal function (1). Degenerative neurons can promote the activation of glial cells (2), especially microglia expressing P2rx7, through the sustained release of ATP (3). Mitochondrial dysfunction can result from a high demand for energy, high levels of ROS generation and unpaired mitochondrial quality control induced by tau pathology, leading to mtDNA leakage and the secretion of mitochondrial-derived vesicles (MDVs) and EVs containing damaged molecules (4). EVs containing damaged mitochondrial molecules activate the inflammatory signaling response in microglia via the recognition of damage-associated molecular patterns (DAMPs; e.g., mtDNA and oxidized proteins) by cytosolic sensors associated with the innate immune response (5). Microglia are phagocytic cells that can uptake and secrete p-tau into the extracellular environment through EVs, a mechanism exacerbated during inflammation, contributing to tau propagation (6). *P2rx7* deficiency improves mitochondrial quality control mechanisms and reduces the amount of mitochondrial cargo in EVs secreted in the brain parenchyma of tauopathy mice, resulting in fewer toxic EVs, lowering the inflammatory response from microglia and reducing the propagation of pathological tau originated from neurons (7). Taken together, these findings indicate that P2RX7 plays an important role in mediating microglial EV secretion, the inflammatory response and tau propagation in tau pathology models. All bar graphs represent the mean ± SEM; *P < 0.05, **P < 0.01 and ****P < 0.0001.

To determine how the mitochondrial molecules identified in PS19 BDEVs may perturb microglial function, we next investigated the molecular changes observed in PS19 BDEVs under *P2rx7* deficiency. Among the 204 mitochondrial proteins identified in BDEVs across the three groups, 49 proteins, including TFAM, HSPE1, SOD2, TOMM40 and PDHB, were significantly upregulated in the PS19 group compared with the WT group, and this change was significantly reversed in the PS19/P2rx7^−/−^group (Fig. 8a). Under stress conditions, mitochondria release double-membrane, electron-dense vesicles containing mitochondrial contents^55^, which have also been observed in the brain parenchyma^56^. As mitochondria-derived vesicles may represent a source of mitochondrial content in the BDEVs pool, we detected an enrichment of a small population of double-membrane, electron-dense vesicles in PS19 BDEVs (3.6%), which was reduced in PS19/P2rx7^−/−^BDEVs (2.8%) (Extended Data Fig. 8a). Single-molecule analysis of individual BDEVs using direct stochastic optical reconstruction microscopy (dSTORM) confirmed significantly greater loading of mitochondrial transcription factor A (TFAM) in Pan-tetraspanins(CD63/CD9/CD81) ^+^ EVs from PS19 mice compared to those from WT mice, with levels markedly reduced in EVs from PS19/P2rx7^−/−^mice (Fig. 8b). Consistent with this finding, mitochondrial D-loop DNA levels were significantly higher in BDEVs from PS19 mice compared to WT controls, an effect that was blunted in the PS19/P2rx7^−/−^group (Fig. 8c), indicating the enrichment of mitochondrial components in PS19 BDEVs and their reduction upon *P2rx7* deficiency. The increased levels of mitochondrial proteins in PS19 BDEVs suggest impaired degradation of dysfunctional mitochondria. Interestingly, *P2rx7* deficiency increased the expression of *Pink1* and *Prkn* in microglia (Fig. 5i, j), which together augment a mitophagy pathway that selectively tags damaged mitochondria with phospho-serine 65 ubiquitin (p-S65-Ub) for degradation^57, 58^. Intriguingly, *P2rx7* deficiency reduced the levels of p-S65-Ub specifically in microglia from PS19 mice, suggesting improved clearance of damaged mitochondria accumulated in the PS19 mouse brain (Fig. 8d and Extended Data Fig. 8b, c).

Finally, to elucidate the role of the P2RX7-EV axis in tauopathy, we analyzed the microglial transcriptomic response to BDEVs isolated from PS19 and PS19/P2rx7^−/−^mice following stereotaxic injection into the cortex and hippocampus of 9.5-month-old C57BL/6 mice, followed by FACS-based microglial isolation and bulk RNA sequencing (Fig. 8e). Principal component analysis of all mapped microglial genes showed clear separation of the three groups: PS19/P2rx7^−/−^ BDEVs, PS19 BDEVs, and saline-injected mice (Fig. 8f). Analysis of DEGs revealed downregulation of genes associated with mitochondria (*Tfam*), DNA-sensing (*Zbp1*) and inflammation (*Irf1, Irf9, Rela*, *Nlrp1b*), along with upregulation of the anti-aging gene Klotho (*Kl*), mitochondrial biogenesis regulator *Ppargc1a* and the mitophagy gene *Pink1* in microglia isolated from PS19/P2rx7^−/−^ BDEV-injected mice compared to those injected with PS19 BDEVs (Fig. 8g and Supplementary Table 9). Strikingly, pathway analysis of differentially expressed genes in microglia revealed that PS19 BDEVs induced cytosolic DNA-sensing and type II interferon (IFN) signaling, suggesting that BDEVs containing mitochondrial DNA (mtDNA) may activate their innate immune response. In addition, microglia in PS19 BDEV-injected mice presented upregulation of oxidative phosphorylation and proteasomal degradation pathways, suggesting mitochondrial dysfunction and clearance of oxidized proteins (Fig. 8h, i). These biological processes were significantly dampened in microglia from PS19/P2rx7^−/−^ BDEV-injected mice (Fig. 8h, i), consistent with scRNA-seq findings showing reduced inflammatory activation in microglia from PS19/P2rx7^−/−^ mice compared to PS19 mice. These findings indicate that P2rx7 deficiency preserves mitochondrial quality control, thereby alleviating EV-induced microglial inflammation in tau pathology.

## Discussion

P2RX7 plays a key role in neuroinflammation associated with both Aβ and tau pathologies^21, 22^. However, its contribution to tau pathology progression via microglial activation remains largely uncharacterized. Here, using scRNA-seq, we show that *P2rx7* is predominantly expressed in microglia and drives inflammatory response in a tauopathy mouse model. *P2rx7* deficiency in PS19 mice improved cognitive performance, preserved synaptic integrity, mitigated brain atrophy, and reduced both tau accumulation and microglial inflammatory activation. Microglial activation and the inflammatory response were linked to increased EV biogenesis and secretion driven by tau accumulation. Notably, *P2rx7* deficiency not only reduced the secretion of tau-containing EVs from microglia *in vivo* but also downregulated genes related to EV biogenesis in both hippocampal tissue and microglia (Fig. 8j). Furthermore, cell type-specific knockdown of *P2rx7* in microglia, but not in astrocytes, suppressed tau propagation *in vivo*. Together, these comprehensive findings highlight the microglial P2RX7-EV axis as a promising therapeutic target to mitigate the progression of tau pathology and neuroinflammation in AD.

Previous studies reported failure of mitochondrial homeostasis as a hallmark event in AD^59, 60^. Tau has been shown to interact directly with mitochondria, leading to dysfunction in both neurons^61, 62^ and microglia^63^. Consistent with these reports, we observed an enrichment of oxidative stress-related gene signatures in the hippocampus of PS19 mice, accompanied by a non–cell autonomous glial response to oxidative stress. These findings indicate mitochondrial dysfunction in the PS19 brain, which was abrogated by *P2rx7* deletion. Our study also highlights the role of mitochondrial molecules in BDEVs in mediating the inflammatory activation of microglia, which is increased in PS19 mice and alleviated by *P2rx7* deletion. BDEVs from PS19 mice are enriched in TFAM and mtDNA, suggesting the presence of TFAM-bound mtDNA structures, which are preferential substrates for activating cGAS-mediated interferon signaling^64^. Our findings suggest a mechanism whereby damaged mitochondrial content propagates neuroinflammation through EV secretion in tauopathy (Fig. 8j). In support of this conclusion, studies in a Down syndrome mouse model revealed elevated levels of mitovesicles in the brain, which are stimulated by mitochondrial damage^56^ and impair long-term potentiation^65^.

Mitochondrial homeostasis relies on a balance between organelle biogenesis and mitophagy. Defects in these processes are frequently observed in neurodegeneration^60^, and uncontrolled mitochondrial stress is closely associated with inflammation, as it can ultimately lead to the release of damage-associated molecular patterns (DAMPs), including eATP, ROS, pathological aggregates, and mtDNA^66, 67^. Our study revealed that EVs deliver DAMPs, potentially originating from tau-accumulating neurons, to microglia, thereby causing inflammatory microglial activation (Fig. 8j). *P2rx7* is highly upregulated in FRM, a transitional microglial cluster representing a shift from a homeostatic state toward an InfM or DAM-IRM phenotype. Our study demonstrates that functional P2RX7 is required for the transition from the FRM state to the InfM state, as *P2rx7* deficiency leads to marked reduction in the InfM population and a modest expansion of DAM and IRM states. P2RX7 may also be involved in mitochondrial dysfunction in neurons and other cell types, as persistent P2RX7 activation promotes excessive Ca^2+^ influx, overloading mitochondrial function and driving dysfunction. A similar mechanism has been observed in monocytes from sepsis patients, where P2RX7 activation results in mitochondrial damage^68^. Overloaded mitochondrial function by P2RX7 activation may also result in oxidization and damage to mitochondrial proteins and mtDNA, which can be loaded into EVs.

Microglia are primary brain innate immune cells that respond to DAMPs stimulation via the activation of pathways such as the NLRP3 inflammasome and cytosolic DNA-sensing pathways (e.g., cGAS-STING), amplifying inflammatory cascades^63, 69–72^. Remarkably, *P2rx7* disruption in tauopathy model mice not only reduced the expression of oxidative stress-associated genes but also decreased the conversion of microglia into the InfM state, which is characterized by the expression of *Nlrp3*, *Il1b*, and other proinflammatory cytokines (Fig. 8j). Notably, FRM microglia in PS19/P2rx7^−/−^ mice presented increased expression of *Pink1* and *Prkn*, key regulators of mitophagy, which may explain the reduced accumulation of damaged mitochondria in microglia, as determined by p-S65-Ub staining. Enhancement of mitophagy increases microglial phagocytosis of Ab, suppresses neuroinflammation and restores cognitive function in an AD mouse model^73^. Intriguingly, intracranial injection of BDEVs from PS19/P2rx7^−/−^ mice increased microglial expression of *Ppargc1a* and *Pink1,* key regulators of mitochondrial biogenesis and quality control, compared to BDEVs from PS19 mice. Furthermore, BDEVs from PS19/P2rx7^−/−^ mice induced increased expression of *Kl*, a gene previously shown to enhance the regulation of antioxidant responses^74^ and modulate microglial activation^75^. Together, these findings underscore the role of P2RX7 in promoting the spread of pathological molecules via EVs in tauopathy mice and suggest that its deficiency attenuate pro-inflammatory microglial activation.

Our study has several limitations. First, although previous reports described increased P2rx7 expression in astrocytes in tauopathy, the limited effect of astrocytic P2rx7 knockout on tau propagation may reflect insufficient knockdown efficiency in our model, highlighting a potential model limitation. Moreover, we observed greater *P2rx7* expression in microglia than in astrocytes, which may explain the more pronounced effects of microglial *P2rx7* deletion. Of note, this study focused on the effects of *P2rx7* deficiency within the CNS, and whether peripheral immune cells also contribute to the observed effects in tauopathy remains to be explored. Our single-cell RNA-seq analysis was limited in assessing neuronal responses to *P2rx7* deficiency, as our dissociation protocol preferentially enriched glial populations, resulting in reduced neuronal representation. Finally, whether P2RX7 directly modulates mitochondrial damage or clearance in response to tau has yet to be fully elucidated, and future studies using human-derived samples will be important to validate the relevance of the microglial response to tauopathy-associated BDEVs.

In summary, these findings demonstrate that *P2rx7* deficiency in PS19 mice restores mitochondrial homeostasis and suppresses microglial inflammatory changes, EV secretion, and tauopathy phenotypes, highlighting the critical role of P2RX7 in mediating tauopathy-related disease phenotypes. Targeting the microglial P2RX7-EV axis provides a protective mechanism against the progression of tauopathy-related neurodegenerative processes.

## Methods

### Animals and genotyping

P2rx7^−/−^ mice (B6.129P2-P2rx7^tm1Gab/J)^ (The Jackson Laboratory, 005576) on a C57BL/6 background were crossed with PS19 tau transgenic mice (The Jackson Laboratory, 008169) on a B6/C3 background expressing human tau 1N4R carrying the FTDP-17-linked P301S mutation to generate PS19/P2rx7^−/−^ mice. WT mice were provided by the National Institute of Aging on a C57BL/6 background and were bred separately from all other transgenic mice. Cell-specific Cre driver mice B6.129P2(C)-*Cx3cr1*^*tm2.1(cre/ERT2)Jung*^/J (*Cx3cr1*^*Cre/ERT2*^; The Jackson Laboratory, 020940) and B6.Cg-Tg(GFAP-cre/ERT2)505Fmv/J (*GFAP*^*Cre/ERT2*^; The Jackson Laboratory, 012849) were bred on a C57BL/6 background. P2rx7^tm1a(EUCOMM)Wtsi^ (*P2rx7*^fl/fl^) mice with loxP sites flanking the critical exons of the gene were crossed with each Cre driver mouse separately to generate *Cx3cr1*^*Cre/ERT2*^:*P2rx7*^fl/fl^ and *GFAP*^*Cre/ERT2*^:*P2rx7*^fl/fl^ mice. For all experiments, male and female mice were used in equal proportions. All mouse care and experimental procedures were approved by the Institutional Animal Care and Use Committee of the Mayo Clinic, Florida. All mice were caged in accordance with their own sex and housed in a barrier facility with a 12 h light and 12 h dark cycle. Food and water were provided ad libitum. Throughout the life of all the mice, veterinary staff closely monitored the animals for complications. The sample sizes for the behavior experiments were based on estimates to provide 80% power to see significant differences of at least 20%. All the mice were deidentified, and the data were analyzed by an experimenter who was blinded to the genotypes of the animals.

### Animal Behavior

#### Contextual and cued fear conditioning

The mice were transferred to the behavior room at least 2–3 hours prior to the experiments for habituation. The tests were conducted in a sound attenuated test chamber with a grid floor capable of delivering an electric shock and overhead cameras to track movement/freezing (freezeframe). On day 1 (training), mice were allowed to explore the chamber for 2 minutes until 80 dB white noise was turned on for 30 seconds (conditioned stimulus, CS), followed by a mild foot shock (.2 mA) (unconditioned stimulus, US) delivered during the last 2 seconds. After 2 minutes, another CS–US pair was administered. Thirty seconds after this final CS–US pairing, mice were returned to their home cages overnight undisturbed. On day 2 (18–24 hours later), mice were returned to the test chamber, and contextual freezing behavior was recorded for 5 minutes. After 2 hours, the environmental and contextual cues were altered by partitioning the chamber with a diagonal divider, red house lights were used outside the chamber instead of white lights, the wire floor was covered with plastic, and vanilla extract was placed in the chamber (on the other side of the divider so that the mouse could not physically interact with it). Freezing behavior was recorded for 3 minutes until the 80 dB white noise CS was presented, and freezing behavior was recorded for an additional 3 minutes. The final 3 minutes refer to the cued fear conditioning phase.

### Cortical atrophy analysis

Mouse brains were coronally sectioned at a thickness of 30 μm starting from bregma +2.1 mm to the dorsal end of the hippocampus at bregma −3.9 mm. Ten coronal sections 300 μm apart from each other were washed in PBS and mounted on negatively charged glass slides for each mouse. Mounted sections were stained with 0.1% Sudan black in 70% ethanol at RT for 20 min and then washed twice in 70% ethanol for 1 min, followed by washing with Milli-Q water for 1 min three times. The stained slices were allowed to dry at RT and mounted with Fluoromount mounting medium (Invitrogen, 00-4958-02). Slides were imaged using Aperio eSlide (Leica), and regions of interest (ROIs) were measured using Aperio Scope software. Volumetric analysis was performed as previously published^76^. Quantification was performed from bregma −1.1 mm to −3.9 mm for the hippocampus and posterior lateral ventricle and from bregma −2.3 mm to −3.9 mm for the piriform/entorhinal cortex. The volume was calculated using the following formula: volume = (sum of area) × 0.3 mm.

### Histological processing and immunofluorescence staining

Brains were harvested following transcardial perfusion with ice-cold PBS, then post-fixed in 4% PFA overnight at 4 °C, except for Cre-inducible mice, which were transcardially perfused with PBS and 4% PFA. The following day, brains were transferred to a cryoprotection solution containing 30% sucrose in PBS and incubated for two days at 4 °C, then embedded in O.C.T. compound (Fisher Scientific, 23-730-571). Coronal sections were cryosectioned at a thickness of 30 μm using a cryostat except for the tau propagation paradigm, where sagittal cryosections were obtained. Sections were washed with PBS for 10 min prior to antigen retrieval in 10 mM sodium citrate at 80 °C for 20 minutes. Following retrieval, sections were washed three times for 5 minutes each in PBS with 0.02% Triton X-100 and incubated with blocking solution (5% normal goat or donkey serum, 5% bovine serum albumin (BSA), and 1% Triton X-100) for 1 hour at RT. Antibody staining buffer consisted of 5% BSA and 1% Triton X-100 in PBS. The following antibodies and reagents were used for immunofluorescence staining: mouse anti-phospho-tau Ser202/Thr205 (1:100; Thermo Fisher Scientific, AT8, MN1020), mouse anti-misfolded-tau (Alz50, 1:50; MC1, 1:400; gift from Peter Davies), mouse anti-human tau (1:1,000; Thermo Fisher Scientific, HT7, MN1000B), rabbit anti-IBA1 (1:1,000; Wako, 019-19741), rat anti-CD68 (1:500; BioLegend, 137001), rabbit anti-PSD-95 (1:500; Abcam, ab18258), guinea pig anti-VGLUT1 (1:1,000; Synaptic Systems, 135304), rabbit anti-GFP (1:500; Novus Biologicals, NB600-308), goat anti-IBA1 (1:1,000; Thermo Fisher Scientific, PA5-18039), rabbit anti-pS65-Ub (1:400; gift from Wolfdieter Springer Lab^58, 77^) and Alexa Fluor secondary antibodies (1:1,000; all from Thermo Fischer Scientific). Primary and secondary antibody staining were performed overnight at 4 °C and for 2 hours at RT, respectively. Following staining, sections were washed with PBS containing 0.02% Triton-X and then mounted with Fluoromount-G with DAPI (Invitrogen, 00-4959-52). Confocal images were taken using SP8 laser confocal microscope with Lightening deconvolution processing (Leica). Images for mEm-CD9 particle analysis were taken using 63× oil immersion/1.4N.A. objective, and 3.69 optical zoom (size is approximately 50 μm by 50 μm) at a pinhole of 1.0 airy unit. Confocal stacks of images with a resolution of 1,024×1,024 pixels were collected using a system-optimized Z-stack interval of 0.25 μm. Processing of Z-stack images, quantification of particles, filaments, volume and colocalization were quantified using IMARIS rendering software (Oxford Instruments). Tau and pTau immunofluorescence analysis and quantification were performed using FIJI software. All the plotted values resulted from the average of 2–3 brain sections per sample.

### Synaptic puncta analysis

VGLUT1 and PSD-95 puncta and overlap analysis were performed using IMARIS software. Briefly, confocal images of the CA1 hippocampal region were acquired using a 63× oil objective as above. On IMARIS, we performed background subtraction of objects larger than 0.5 mm in diameter and count the surfaces for VGLUT1 and PSD-95 filtering by puncta volume in the range of 0.1–0.8 mm^3^ to exclude noise or larger objects to be considered synapses^78^. For VGLUT1 and PSD-95 colocalization, we counted overlapping puncta by setting a maximum distance <0.151 mm between both surfaces.

### Biochemical sequential extraction of sarkosyl-insoluble fractions from mouse brain tissues

Sarkosyl-insoluble tau fractions were isolated from temporal cortex brain tissues from mice by sequential centrifugation, as previously reported^16^. Briefly, mice were transcardially perfused with PBS, and the brain tissue was snap-frozen on dry ice and stored at −80 °C until protein extraction. Temporal cortex tissue was dissected and homogenized in ten volumes of TBS buffer (50 mM Tris-Cl, pH 8.0 in saline) supplemented with halt protease and phosphatase inhibitor cocktail (Thermo Fisher Scientific, 78442). The tissue homogenate was centrifuged at 48,300*g* for 20 min at 4 °C. The supernatant and pellet were designated as S1 (TBS-supernatant) and P1 (TBS-pellet) fractions, respectively. The S1 fraction was ultracentrifuged at 186,000*g* for 40 min at 4 °C, and the pellet (S1p) was resuspended in 20 μl of PBS. The P1 fraction was resuspended in five volumes of wet weight of the original tissue using buffer B (1% sarkosyl, 10 mM Tris, pH 7.4; 800 mM NaCl; 10% sucrose; 1 mM EGTA; and 1 mM PMSF, all from Sigma–Aldrich) and incubated with a benchtop thermomixer at room temperature for 1 h. The samples were ultracentrifuged at 186,000*g* for 1 h at 4 °C. The sarkosyl-insoluble pellet (P2) was resuspended in 20 μl of PBS. Both the S1p and P2 pellets were stored at −80 °C until use.

### ELISA

TheS1p and P2 fractions were diluted 1:10 in 8 M guanidine buffer for tau solubilization for 3 h at room temperature with agitation. Samples were sonicated (0.15 hertz pulses with 5 second durations repeated three times) and diluted in TENT buffer (50 mM Tris HCl pH 7.5, 2 mM EDTA, 150 mM NaCl, and 1% TritonTM X-100) supplemented with a halt protease and phosphatase inhibitor cocktail. Samples were subjected to human total tau (Invitrogen, KHB0041) and pTau-Ser396 ELISA (Invitrogen, KHB7031) according to the manufacturer’s instructions.

### Bulk RNA sequencing and analysis

Hippocampal tissues from 9.5-month-old WT (n=8), P2rx7^−/−^ (n=8), PS19 (n=10) and PS19/P2rx7^−/−^ (n=6) mice, including an equal distribution of males and females per group, were homogenized in 700 μL of QIAzol lysis reagent. Total RNA extraction was performed using miRNeasy Plus Micro Kit (Qiagen, 217084) according to the manufacturer’s protocol for the purification of total RNA. cDNA library construction and sequencing were performed via the Genome Analysis Core at the Mayo Clinic Rochester on the NovaSeq X platform, with a sequencing depth of 100 million total reads per sample. Raw RNA sequencing paired-end reads were processed through the Mayo RNA-Seq bioinformatics pipeline, MAP-RSeq version 3.1.4^79^. Briefly, MAP-RSeq employs the fast, accurate and splice-aware aligner STAR^80^ to align reads to the reference mouse genome build mm10. Gene and exon expression quantification was performed using the Subread^81^ package to obtain both raw and normalized reads. Finally, comprehensive analyses were run on the aligned reads, confirming the quality of the sequenced libraries. RNA-seq analysis was performed using Omics Playground (BigOmics) platform. Briefly, genes with low expression were filtered out, and the raw read counts were normalized using log2CPM+quantile normalization method. Differential expression analysis was performed using three independent statistical methods: DESeq2 (Wald test), edgeR (QLF test) and limma-trend. The maximum *q*-value of the three methods was taken as the aggregated *q*-value, which corresponds to the intersection of significant genes from all three tests. PCA plots were generated via ClustVis (https://biit.cs.ut.ee/clustvis/).

### Tamoxifen administration

Tamoxifen solution (Sigma-Aldrich, T5648–5G) was prepared in corn oil at 40 mg/ml by shaking overnight at 37 °C. For Cre inducible deletion of *P2rx7* in a cell type-specific manner, starting at 2 months of age, *Cx3cr1*^*Cre/ERT2*^:*P2rx7*^fl/fl^ and *GFAP*^*Cre/ERT2*^:*P2rx7*^fl/fl^mice were randomized to receive either 100 mg/kg body weight tamoxifen or corn oil injection intraperitonially once a day for a total of 5 consecutive days. During the study, both groups gained weight normally, and no adverse effects were observed in the animals that received either treatment.

### Stereotaxic surgery

All stereotaxic injections were performed in accordance with the Mayo Clinic Institutional Animal Care and Use guidelines. Mice were deeply anesthetized with 1 to 2% isoflurane in a 95% O2 and 5% CO2 mixture. Injections were performed via a computer-controlled stereotaxic frame (Neurostar, Germany) equipped with a glass needle filled with virus, except for the mEm-CD9 tracking paradigm, where a manual stereotaxic apparatus was used. The needle was slowly lowered to the target site at an insertion speed of 0.1 mm/sec, the injection was performed at a rate of 200 nL/min, and the needle remained at the target site for 5 min after the injection. To study the effects of microglial and astrocytic cKO of P2rx7 on tau propagation, *Cx3cr1-CreERT2-P2rx7*^*fl/fl*^ and *Gfap-CreERT2*-P2rx7^fl/fl^ mice at 3 months of age were unilaterally injected with 0.7 μL of AAV2/6-SYN1-P301L tau (3.89E+12 GC/ml, plasmid packaged in Boston Children’s Hospital) into the right MEC (AP: −4.85, ML: +3.45, DV: 3.30 mm from the skull) and incubated for 4 weeks before being euthanized for immunohistochemical analysis. For mEm-CD9 particle tracking *in vivo*, C57BL/6 and P2rx7^−/−^ mice at 3 months were first unilaterally injected with 1 μL of pLV-EF1α-mEmerald-CD9-miR9T lentiviral particles (viral titer: >10^9^ TU/ml) and injected 2 weeks later with 0.7 μL of AAV2/6-SYN1-P301L tau into the hippocampus (AP: −2.18 mm; ML: 1.9 mm; DV: −1.3 mm). After 4 weeks, the mice were euthanized for immunohistochemical analyses. To explore the effect of BDEVs on the microglial response, 3 μL (1E^9^ particles total) of PS19-EVs, PS19/P2rx7^−/−^-EVs or saline control were injected into the cortex (AP: −2 mm; ML: ±1.5 mm; DV: ±0.8 mm) and hippocampus (AP: −2 mm; ML: ±1.5 mm; DV: ±2 mm) bilaterally. After 16 h, microglia were isolated from the brain regions surrounding the injection sites, followed by RNA-seq analysis.

### Microglia isolation for RNA-seq

Microglial isolation was performed as previously published^82, 83^. Briefly, mice were euthanized in a CO2 chamber and transcardially perfused with ice-cold Hanks’ balanced salt solution (HBSS; Thermo Fisher, 14175103). Brain regions around the injection sites were dissected and minced into small pieces. The tissue was gently homogenized using a Dounce homogenizer and centrifuged at 350*g* for 5 min at 4 °C. The cells were resuspended in 37% Percoll Plus (GE Healthcare, 17-5445-02) in HBSS, loaded on top of a 70% Percoll layer and centrifuged at 800*g* for 25 min at 23 °C with acceleration set to 3 and deceleration set to 1. Three milliliters of cells were aspirated from the interface layer and washed with HBSS by centrifugation at 350*g* for 5 min at 4 °C. Cells were stained with rat allophycocyanin (APC)-conjugated anti-mouse FCRLS (1:1,000; clone 4G11, gift from Butovsky Lab^84^), phycoerythrin (PE)-Cy7-conjugated anti-mouse CD11b (1:300; eBioscience, 50-154-54) and PerCP/Cy5.5-conjugated anti-mouse Ly6C (1:300; Biolegend, 128012) in 0.2% BSA in HBSS on ice and protected from light for 15 min. After being washed, the cells were incubated with SYTOX blue (1:150, Thermo Fisher Scientific, S34857), and SYTOX^−^Ly6C^−^CD11b^+^FCRLS^+^ live microglia were sorted using a Bigfoot Cell Sorter (Thermo Fisher Scientific), followed by RNA isolation and cDNA preparation using SMART-seq v4 Ultra Low Input RNA Kit protocol. cDNA library construction and sequencing were performed via the Genome Analysis Core at the Mayo Clinic Rochester as described above.

### Single-cell dissociation from brain tissue

For single-cell preparations, mice were transcardially perfused with PBS, and brains were immediately transferred to ice-cold PBS. On a Petri dish, cerebellum was dissected and discarded, and the remaining brain tissue was sliced into 8 sagittal pieces. Tissue dissociation was performed using the Adult Brain Dissociation Kit (Miltenyi Biotec, 130-107-677) and gentle MACS Octo Dissociator, according to the manufacturer’s recommendations. After tissue dissociation, the cell suspension was filtered through a 70-μm MACS SmartStrainer (Miltenyi Biotec, 130-110-916) and centrifuged at 300*g* for 10 minutes at 4 °C. The cell pellet was fully resuspended in 4 mL of 1:4.5 debris removal solution (Miltenyi Biotec, 130-109-398) diluted in PBS and gently overlaid with 4 mL of PBS. The solution was centrifuged at 3,000*g* for 10 minutes at 4 °C with the acceleration set to 3 and the brake set to 0. The supernatant, including the myelin layer disc, was discarded, and 15 mL of PBS was added to the top of the cell pellet. Tubes were gently inverted three times and centrifuged at 1,000*g* for 10 minutes at 4 °C. Finally, the total brain cell pellet was resuspended in 300 μL of 0.2% BSA in PBS and processed for single-cell RNA sequencing or for sorting of microglia and astrocytes using CD11b (Miltenyi Biotec, 130-093-636) or ACSA2 (Miltenyi Biotec, 130-097-679) magnetic beads, respectively.

### RT–qPCR

Total RNA was isolated from sorted microglia and astrocyte cells using the miRNeasy Plus Micro Kit (Qiagen, 217084) and reverse transcribed to complementary DNA sequences via the SuperScript IV VILO Kit (Invitrogen, 11756050). Quantitative PCR was performed using Taqman probes with TaqMan Fast Advanced Master Mix (Applied Biosystems, 4444557).

### Single-cell RNA sequencing

Single-cell suspensions for single-cell RNA sequencing were generated from 9.5-month-old WT (n=4), PS19 (n=4) and PS19/P2rx7^−/−^ (n=4) mice, including an equal distribution of males and females per group, as described above, with minor adaptations. Briefly, 5 μg/mL actinomycin D (Sigma–Aldrich, A1410) was added to ice-cold PBS for perfusion and brain storage buffers for transcription inhibition. For tissue dissociation, 5 μg/mL actinomycin D, SUPERase In RNAse inhibitor 1:1,000 (Thermo Fischer Scientific, AM2694) and 1 U/mL DNAse I (Whorthington Biochemical, LS006333) were added to the enzyme dissociation solution. The single-cell suspension was immediately processed using the Evercode^™^ Cell Fixation V3 Kit (Parse Biosciences, ECFC3300) and then cryopreserved at 1,000 cells/μL until library preparation. Samples were processed and fixed in batches, each comprising four samples. Library preparation was performed in a single batch. Eight sublibraries of 12,500 cells each (total: 100,000 cells) were generated using the Evercode WT V3 Single-Cell Whole Transcriptome Kit (Parse Biosciences, ECWT3300), which utilizes split-pool combinatorial barcoding and instrument-free technology for single-cell transcriptome sequencing, according to the manufacturer’s protocol. The cDNA library concentration was determined using the Qubit dsDNA HS Assay Kit, and cDNA quality was assessed using the Bioanalyzer High Sensitivity DNA Analysis Kit (Agilent, 5067–4626) on a 2100 Bioanalyzer System (Agilent). Libraries were sequenced using the Illumina NovaSeq X Plus platform at the Genome Analysis Core at Mayo Clinic Rochester for a sequencing depth of 50,000 read pairs/cell.

### Single-cell RNA sequencing processing and analysis

#### Data processing

The raw reads sequencing files were processed using the automated pipeline from Parse Biosciences on Trailmaker software (Parse Biosciences) for barcode correction, read alignment, read deduplication, and transcript quantification to generate a cell-by-gene count matrix. The generated count matrices were analyzed using Trailmaker insight module for data processing and quality control. To filter low-quality cells, we removed cells with lower transcripts on the basis of a data-dependent threshold via the cell size distribution filter and cells that contained greater than 15% mitochondrial genes. Cells deviating from the linear relationship between the number of genes versus the number of transcripts were removed. Doublets were filtered out via scDblFinder. Data integration was performed via Harmony using 2,000 top highly variable genes and the log normalization method. Twenty-two principal components that explained >90.4% of the variation were used for dimensionality reduction before UMAP embedding, with a minimum distance= 0.3 using Cosine metric and Leiden clustering method, with resolution= 0.8.

#### Microglia subclustering and trajectory analysis

For the final analysis of microglia, we combined microglial and immune cell clusters to filter out nonmicroglia (macrophage, neutrophil, B and T-cells) and low-quality clusters.

After filtering, 22,836 microglial cells were retained for downstream analysis. Using the previously integrated dataset, we selected 32 principal components for dimensionality reduction by UMAP with a minimum distance= 0.018 using Cosine metric and Leiden clustering method with resolution= 0.3. Trajectory analysis was performed in Trailmaker using the Monocle 3 workflow. To infer how homeostatic microglia transition into disease cell states, after the calculation of root nodes, we set the initial roots that overlap with the most homeostatic cell cluster to perform pseudotime analysis.

### Pathway analysis

The top-ranked DEGs and DEPs (*P* < 0.05) were used to perform pathway analysis. Pathway analysis of the proteomics and scRNA-seq datasets were performed via Metascape (http://www.metascape.org)^78^ and ShinyGO 0.82 (https://bioinformatics.sdstate.edu/go/)^85^. GSEA was performed via Omics Playground (BigOmics) using *q*-values yielded by the different statistical methods combined into a meta-*q* value for the gene set enrichment score and the generation of the gene set fold-change, which was defined as the average of the fold-change values of its member genes. DEGs with corresponding fold changes and adjusted *P* values were subjected to Ingenuity Pathway Analysis (https://digitalinsights.qiagen.com/products-overview/discovery-insights-portfolio/analysis-and-visualization/qiagen-ipa/) for canonical pathway analysis and prediction of master regulators of the identified biological networks. The protein interaction network was visualized via STRING 12.0 (https://string-db.org/)^86^.

### Isolation of extracellular vesicles from mouse brain tissue

EVs were isolated from unfixed and snap-frozen hemi-brain tissue (~0.3 g) from mice transcardially perfused with PBS based on our previous method with minor modifications^14, 52, 54, 87^. The tissue was sliced with a razor blade into small sections 2−3 mm3 on dry ice and then dissociated in 3 mL of Hibernate-E medium (Thermo Fisher Scientific, A1247601) containing 75 U/mL collagenase type 3 (Worthington Biochemical, CLS-3, LS004180) at 37 °C for 15 min, with gentle shaking every 5 minutes. Enzymatic digestion was immediately stopped by the addition of 9 mL of ice-cold Hibernate-E solution containing halt protease and phosphatase inhibitor cocktail (1:100). The solution was filtered through a 40-μm mesh filter (Thermo Fisher Scientific, 22-363-547) and sequentially centrifuged at 300*g* for 15 min at 4 °C to remove cells, 2,000*g* for 15 min at 4 °C to remove membrane debris, and then 5,000*g* for 30 min at 4 °C. The supernatant was filtered through a 0.45-μm pore-size syringe filter (Millipore, SLHPM33RS) into 13.2 mL polypropylene (Beckman Coulter, 331372) to deplete tissue debris and large vesicles and ultracentrifuge at 100,000*g* for 70 min at 4 °C using a swing bucket rotor (SW 41 Ti, Beckman Coulter). The pellet was resuspended in 2 mL of 0.475 M sucrose (Thermo Fisher Scientific, S5–3) in double-filtered PBS (dfPBS; 0.22-μm pore-size syringe filter) and then overlaid on top of five sucrose cushion layers (2 mL each starting from 2.0 M, 1.5 M, 1 M, 0.825 M and 0.65 M in dfPBS) for density separation of EV-enriched fractions by ultracentrifugation at 200,000*g* for 20 h at 4 °C. Two 2 mL EV-enriched fractions were collected from the interphases between the 0.65 and 0.825 M fractions and the 0.825 M and 1.0 M fractions, combined with 8 mL of dfPBS and ultracentrifuged at 100,000*g* for 70 min at 4 °C. The final purified brain-EV pellet was resuspended in 30 μL of dfPBS and stored at −80 °C.

### Nanoflow cytometry (NanoFCM)

The size and concentration of BDEVs were analyzed using nanoflow cytometry (Flow NanoAnalyzer, NanoFCM). After calibration using concentration and small size beads, 20 μL of each diluted BDEV sample (1:200) was initially loaded and boosted for 45 s at 1 kPa pressure, followed by 1 min of data acquisition.

### Cryo-EM analysis

EV samples were diluted 1:10 in 0.1 M phosphate buffer. Grids (Quantifoil R2,2 300 mesh, EMS) were charged for 30 seconds with a Pelco EasiGlow glow discharge cleaning system (Ted Pella). A total of 4.5 μL of sample was applied to the grid, which was then blotted and plunge-frozen using a Vitrobot IV (FEI). The Vitrobot was set to 22 °C and 100% humidity, the blot time was 4.5 s, the wait time was 5 s, the blot force was 2 (AU), the blot total was set to 1, and the drain time was set to 0 s. EV samples were loaded into the TEM using a Gatan 626 single tilt cryo-EM holder. Images were taken with a Talos L120C transmission electron microscope (Thermo Fisher Scientific) equipped with a BM-Ceta metal-oxide semiconductor camera. Images were acquired at 120 kV at magnifications of 28,000x and 57,000x. Images were taken via TEM Image and Analysis (TIA) software. EV size was manually measured using FIJI software.

### Protein extraction, concentration and western blotting

Brain tissue and EV samples were lysed in cold RIPA buffer (Thermo Fisher Scientific, 89900) supplemented with halt protease and phosphatase inhibitor cocktail (and sonicated for 5 min). A Micro Bicinchoninic acid (micro-BCA) assay (Thermo Fisher Scientific, 23235) was used to determine the protein concentration of each sample.

#### Western blotting

Five micrograms of brain lysates and EVs were denatured in Laemmli sample buffer (Bio-Rad, 1610737), loaded on 4 to 20% Tris-Glycine SDS–PAGE gels (Bio-Rad, 4561096), and then electrotransferred to 0.45-μm nitrocellulose membranes (Bio-Rad, 1620115). The membranes were blocked with 5% nonfat milk (Cell Signaling Technology, 9999S) in PBS and incubated with primary antibodies overnight at 4 °C. The following primary antibodies were used for immunoblotting: anti-CD81 (1:300, BioLegend, 104902), anti-GM-130 (1:100, Santa Cruz Biotechnology, sc55591), anti-Alix (1:500, Abcam, ab76608), anti-EEA1 (1:100, Santa Cruz Biotechnology, sc-365652), anti-annexin A2 (1:500, Abcam, ab178677), and anti-β-actin (1:500, Santa Cruz Biotechnology, sc-47778). The membranes were further incubated with appropriate horseradish peroxidase (HRP)-labeled secondary antibodies (anti-mouse IgG, HRP-linked antibody; Cell Signaling Technology, 7076S; anti-rabbit IgG, HRP-linked antibody; Cell Signaling Technology, 7074S; anti-Armenian hamster IgG, HRP-linked antibody; Santa Cruz Biotechnology, sc-2789) for 1 hour, and immunoreactivity was captured using enhanced chemiluminescence solutions (Millipore, WBKLS0100). All the images were detected using the ChemiDoc MP Imaging System (Bio-Rad).

### BDEV proteomics

Equal amounts of BDEV lysates (4.5 μg) from WT, PS19 and PS19/P2rx7^−/−^ mice (n=5 mice per group) were prepared for data independent acquisition (DIA) mass spectrometry at Mass Spectrometry facility at UMass Chan Medical School.

#### Sample preparation

The samples were flash frozen on dry ice and dried with a Speed-Vac. A total of 23 μl of 1x lysis buffer was added to the protein pellet, which was started following the suspension trap (S-Trap) protocol. The proteins were reduced by TCEP and alkylated by iodoacetamide. After overnight digestion at 37 °C, 40 μL of 50 mM TEAB elution buffer was added, and the mixture was spun at 4,000 rpm for 1 min, followed by a 1 min spin with 40 μL of 0.2% formic acid (FA) and lastly 140 μL of 50% acetonitrile (ACN). The elution was then sped-vac to dry. The digested samples were reconstituted with 20 μL of 0.1% FA in 5% acetonitrile and centrifuged at 16,000*g* for 15 min. Eighteen microliters of each sample were transferred to nonbinding HPLC vials.

#### LC–MS/MS Analysis

The samples (4 μL injection) were analyzed on a TimsTOF Pro (Bruker) mass spectrometer coupled to a nanoElute (Bruker) LC system. A Bruker Fifteen Pepsep column (150 mm × 75 μm id, 1.9 μm, 120 Å pore size) was used for all the analyses. A 60-minute gradient was used for DDA-PASEF, and a 30-minute gradient was used for DIA-PASEF analysis. Other parameters were the same for all analyses, including flow rate (500 nL/min), capture nanoelectrospray voltage (1600 V), column configuration (notrap-elute) and solvent composition (Solvent A: 0.1% FA in water and Solvent B: 0.1% FA in ACN).

DIA-PASEF method: Samples were acquired with a method consisting of 22 mass width windows (40 Da width, from 275 to 1155 Da) with 1 mobility window each covering the ion mobility range (1/K0) from 0.70 to 1.30 V s/cm2. These windows were optimized with the Window Editor utility from the instrument control software (timsControl, Bruker) using one DDA-PASEF run acquired from the analyzed samples.

#### Data analysis workflows using Spectronaut, library-free (directDIA+)

The directDIA+ workflow in Spectronaut (v18.2) software was used for analyzing the DIA-PASEF dataset, with no need to build a library from the DDA runs. First, the raw files were converted to HTRMS files, and the data were searched against a reviewed mouse (UP000000589) isoform fasta database (downloaded on 08/30/2023, containing 21837 sequences). The default factory settings were used for the Pulsar search and DIA analysis (including Trypsyin/P as the enzyme; 7–52 peptide length range, up to two missed cleavages allowed; oxidation of Me and acetylation of protein N-t as variable modifications; carbamidomethyl of C as a fixed modification; and 1% FDR for PSM, peptide and protein identification). Automatic cross-run normalization and the protein LFQ method were used for protein quantification, and protein quantity was determined at the MS2 level via the area of extracted chromatogram traces.

#### *Differential expression analysis of the* BDEV *proteome*

Differentially expressed proteins (DEPs) in BDEVs were compared via Student’s t test (*P* < 0.05) among the 1) PS19 and WT groups, 2) PS19/P2rx7^−/−^ and WT groups and 3) PS19/P2rx7^−/−^ and PS19 groups. Pairwise comparisons with corresponding *P* values and fold changes are provided in the Supplementary Materials.

### DNA isolation from BDEVs and mtDNA qPCR

For total DNA isolation from BDEVs, 2.5 μL of BDEVs were treated with DNAse (1 U) (Thermo Fisher Scientific, EN0521) in 10 μL of solution for 30 min at 37 °C, followed by the addition of 1 μL of 50 mM EDTA and heat inactivation for 10 min at 65 °C. BDEV lysis and protein kinase digestion were performed using the QIAamp DNA Micro Kit (Qiagen, 56304) according to the manufacturer’s instructions. The DNA was purified by adding 0.8 μL AMPure XP magnetic beads (Beckman Coulter, A63880) to 1 μL of EV lysate. Total DNA was resuspended in nuclease-free water. qPCR for detection of the mtDNA *Dloop* gene was performed by mixing 1.5 ng of total EV DNA, 400 nM *Dloop* primers (forward primer: AGGTTTGGTCCTGGCCTTAT and reverse primer: GTGGCTAGGCAAGGTGTCTT) and 12.5 μL of SYBR Green Universal Master Mix (Applied Biosystems, 4309155) in 25 μL of solution. The cycling threshold (Ct) values obtained for mtDNA in WT-EVs were used as normalization controls for mtDNA values.

### Single-molecule super-resolution microscopy

We performed single-molecule imaging of mitochondrial TFAM molecules in BDEVs using dSTORM. On day 1, 2.5E8 EV particles were mixed with Alexa-647-conjugated TFAM (1:200, anti-mouse Cell Signaling Technology, 8076S) and FITC-conjugated Pan-tetra antibodies (1:8, anti-mouse CD63, Biolegend, 143920; 1:8, anti-mouse CD9, Biolegend, 124808; 1:8, anti-mouse CD81, Thermo Fisher Scientific, MA5–17939) in 8 μL of PBS-0.1% Triton-X/1% casein/5% FcR blocking (Miltenyi Biotec, 130-092-575) and incubated in the dark overnight at 4 °C. For EV capture, we prepared custom coverslips by adding a 10 μL droplet of a 4% solution of MPTS (3-mercaptopropyl trimethoxy silane, 95%) dissolved in ethanol to the cleaned glass coverslips and incubated them for 30 minutes at room temperature to form a silane-based layer on the surface. Thereafter, the coverslips were washed with ethanol to remove any unbound MPTS and were dried for less than 5 minutes. Next, each coverslip was treated with a 5 μL droplet of GMBS (Sulfo-GMBS (N-γ-maleimidobutyryl-oxysulfosuccinimide ester)) dissolved in dfPBS for 30 minutes to facilitate antibody conjugation. Coverslips were washed three times with 1 mL of dfPBS and incubated with 5 μL of the capture antibody TIM4 (anti-mouse, Fujifilm, 137–18511) in the dark overnight at 4 °C. On day 2, 1.25 μL of calcium chloride (CaCl2) solution was added to the labeled EV particles prior to loading onto the capture coverslip, and the mixture was incubated at room temperature for 1 hour to ensure immobilization of the EVs on the modified coverslips. Coverslips were washed three times with 1 mL of dfPBS and fixed by adding 5 μL of 4% paraformaldehyde/4% sucrose in dfPBS for 10 minutes. The fixative agents were washed with dfPBS, and coverslips were mounted after adding 5 μL of BCubed Imaging Buffer (ONI, 900–00004). We applied dSTORM using the super-resolution microscope Nanoimager (ONI) equipped with a 100X oil objective (1.4 numerical aperture). Fluorescence images were acquired under 60% power on a 640 laser channel and 35% power on a 473 laser, and a total of 3,000 frames (1,500 each) were captured using a 53.5° total internal reflection fluorescence (TIRF) illumination angle to obtain the optimal signal/noise ratio. Images were processed and analyzed via the CODI online platform from ONI (https://alto.codi.bio/), including filtering and drift correction. Clusters with at least 3 individual molecules were used for analysis.

### Weighted gene co-expression network analysis (WGCNA)

The WGCNA algorithm was used to define gene and protein co-expression networks on the basis of normalized genes in RNA-seq or protein abundance of BDEVs datasets using R (version 4.4.0), as previously described^43, 54^. Briefly, genes or proteins were hierarchically clustered via the calculation of topological overlap with a bicor correlation function. Module assignments were determined via dynamic tree-cutting with the following specified parameters: soft threshold power beta = 14 (bulk RNA-seq) and 15 (proteomics), deepSplit = 2, minModulesize = 30, merge cut height = 0.20 (bulk RNA-seq) and 0.25 (proteomics), unsigned (bulk RNA-seq) and signed (proteomics) network with partitioning of medoids with respect to the dendrogram, and a reassignment threshold of *P* < 0.05. A total of 17 modules of co-expressed genes in hippocampal RNA-seq data and 22 modules of co-expressed proteins in BDEVs were generated to define the eigengene/eigenproteins (1st principal component of the module), which represent the most representative abundance value for a module and explain the covariance of all proteins within a module. In addition, Pearson correlations as well as the correlation significance between module eigengenes/eigenproteins and each trait (fear conditioning, hippocampus volume, piriform/entorhinal cortex volume and insoluble pTau-Ser396) were generated.

### Statistical analysis

Sample sizes were chosen on the basis of standard power calculations (with a power of 0.8 and α = 0.05) and similar experiments that were previously published. In general, statistical methods were not used to recalculate or predetermine sample sizes. Animals from different cages but within the same experimental group were selected to ensure randomization. Data acquisition and analysis were performed by different experimenters blinded to the genotypes of the animals. The ROUT test was used to identify and discard potential outliers without any prior exclusion criteria. All data in the graphs are presented as the mean ± standard error of the mean (SEM). Statistical analyses were performed using GraphPad Prism v10.4.1 (GraphPad Software), and R v4.4.0 was used for WGCNA. Two-group comparisons were made via two-tailed unpaired Student’s t test. For multiple comparisons, one-way ANOVA with Holm–Sidak’s test or the Kruskal–Wallis test with the Wilcoxon test was used, as indicated in the figure legends and methods section. For differential expression analysis of transcriptomic data, significance was defined as a FDR-corrected *P*-value (*q*-value).

## Supplementary Material

Supplementary Files

This is a list of supplementary files associated with this preprint. Click to download.

• SupplementaryFig.1.UncroppedimagesforimmunoblotsassociatedwithFig.7c.pdf

• SupplementaryFig.2.Figureexemplifyingthegatingstrategyformousemicrogliafl uorescenceactivatedcellsorting.pdf

• SupplementaryTable1.HippocampusbulkRNAseq.xlsx

• SupplementaryTable2.scRNAseqofbraincellclustersPS19vsWTmice.xlsx

• SupplementaryTable3.scRNAseqofbraincellclustersPS19P2rx7KOvsPS19mice.xlsx

• SupplementaryTable4.scRNAseqofglialcellscommomDEGsPS19P2rx7KOvsPS19mice.xlsx

• SupplementaryTable5.scRNAseqofmicrogliaPS19vsWTmice.xlsx

• SupplementaryTable6.scRNAseqofmicrogliasubclustersmaingenesvsallotherclusters.xlsx

• SupplementaryTable7.scRNAseqofmicrogliasubclustersPS19P2rx7KOvsPS19mice.xlsx

• SupplementaryTable8.BrainEVproteomics.xlsx

• SupplementaryTable9.RNAseqofsortedmicrogliafromEVinjectedmice.xlsx

• ExtendeddatafiguresandtablesBodart.pdf

## Data Availability

All the data generated in this study are available in the supplementary information. RNA-seq data will be deposited in the Gene Expression Omnibus, and the LC–MS proteomics data will be deposited in the ProteomeXchange Consortium repository. Accession identifiers and public access will be provided in the final published version of the manuscript.
